# A Comparison of the Pac-X Trans-Pacific Wave Glider Data and Satellite Data (MODIS, Aquarius, TRMM and VIIRS)

**DOI:** 10.1371/journal.pone.0092280

**Published:** 2014-03-21

**Authors:** Tracy A. Villareal, Cara Wilson

**Affiliations:** 1 Marine Science Institute and Department of Marine Science, The University of Texas at Austin, Port Aransas, Texas, United States of America; 2 Environmental Research Division, Southwest Fisheries Science Center, National Marine Fisheries Service, NOAA, Pacific Grove, California, United States of America; Pacific Northwest National Laboratory, United States of America

## Abstract

Four wave-propelled autonomous vehicles (Wave Gliders) instrumented with a variety of oceanographic and meteorological sensors were launched from San Francisco, CA in November 2011 for a trans-Pacific (Pac-X) voyage to test platform endurance. Two arrived in Australia, one in Dec 2012 and one in February 2013, while the two destined for Japan both ran into technical difficulties and did not arrive at their destination. The gliders were all equipped with sensors to measure temperature, salinity, turbidity, oxygen, and both chlorophyll and oil fluorescence. Here we conduct an initial assessment of the data set, noting necessary quality control steps and instrument utility. We conduct a validation of the Pac-X dataset by comparing the glider data to equivalent, or near-equivalent, satellite measurements. Sea surface temperature and salinity compared well to satellite measurements. Chl fluorescence from the gliders was more poorly correlated, with substantial between glider variability. Both turbidity and oil CDOM sensors were compromised to some degree by interfering processes. The well-known diel cycle in chlorophyll fluorescence was observed suggesting that mapping physiological data over large scales is possible. The gliders captured the Pacific Ocean’s major oceanographic features including the increased chlorophyll biomass of the California Current and equatorial upwelling. A comparison of satellite sea surface salinity (Aquarius) and glider-measured salinity revealed thin low salinity lenses in the southwestern Pacific Ocean. One glider survived a direct passage through a tropical cyclone. Two gliders traversed an open ocean phytoplankton bloom; extensive spiking in the chlorophyll fluorescence data is consistent with aggregation and highlights another potential future use for the gliders. On long missions, redundant instrumentation would aid in interpreting unusual data streams, as well as a means to periodically image the sensor heads. Instrument placement is critical to minimize bubble-related problems in the data.

## Introduction

Oceanography requires sampling an environment that covers 70% of the Earth’s surface and has traditionally been done from relatively slow-moving (<10–15 knots) research vessels. The extended oceanographic expeditions of the late 19^th^ and early 20^th^ century lasting months to years [1] have generally been replaced by focused, relatively short duration trips of days to weeks starting in the mid-20^th^ century [2]. While there is often no substitute for collecting water samples, technological developments now permit the deployment of ocean observing systems that generate many types of data collected remotely via electronic or optical sensors that are either stored or transmitted directly to shore [3], [4]. Satellite remote sensing has transformed oceanography by permitting nearly synoptic global measurements of surface parameters ranging from chlorophyll biomass to salinity, and long-term moorings now permit time-series information for the water column at fixed stations.

An additional class of sampling platform that combines mobility with remote operation is underwater unmanned vehicles (UUVs). These are mobile, unmanned submersible systems with sensors that may either be tethered with surface derived power (remotely operated vehicles) or completely autonomous platforms (autonomous underwater vehicles, AUVs) that independently control speed, direction, and depth based on programming in their computers.

Autonomous vehicles provide a number of unique capabilities for sampling the oceanic environment. Their low cost compared to research vessels allows them to be deployed in tandem and sample repeatedly over defined areas yielding high resolution 3-D data sets. For example, a recent analysis found that AUV costs averaged $998 per day while research vessel costs were > $25,000 per day [5] with UNOLS global class vessels exceeding $30,000 per day [6]. Rapid technological developments now permit sophisticated instrumentation to be deployed with telemeter relaying data to shore in near-real time, and when coupled to GPS navigation systems, quasi-independent operation with adaptive sampling. Applications can range from sniffing out unexploded ordinance [7] to tracking and mapping penguin feeding grounds [8]. When equipped with high resolution cameras, long-term monitoring of benthic environments is possible yielding essential information for ecosystem-based fisheries management [9]. Autonomous vehicles are now operational in ocean observing systems with homing and self-docking capability as well as in supporting research vessel operations [10], [11]. They are an essential part of the oceanographic tool box and are rapidly evolving in response to the needs of climate change research [11], particularly in the challenging environment of the Arctic and Antarctic [12], [13]

There are over 50 types of AUVs, based on their speed, propulsion and sampling capability [14]. As independent vehicles, power management is a significant concern. Trade-offs between power requirements and duration have led to several general classes of AUVs defined at one end of the spectrum by systems that have significant battery capacity, relatively high speed and the ability to support energy-intensive sampling equipment and at the other end by more passive propulsion systems, lower battery capacity, slower speeds but much greater endurance. Several propulsion systems that either use changes in ballasting [15], solar power [16], [17], or wave driven propulsion [18] have resulted in significant advances in endurance. Often termed “gliders”, these ultra-low power requiring platforms are capable of extended missions. Trans-Atlantic sampling is now possible, demonstrating the potential for supporting both systematic, autonomous data collection [5], [19] as well as enhanced educational experiences for linked graduate/undergraduate classes [19]. Extended deployments (3 months) of SeaGliders [20] were used to document diatom aggregation after the N. Atlantic spring bloom by changes in fluorescence spiking over a 0–1000 m depth interval [21]. Gliders were also a component of the cleanup of the *Deep Horizon* oil spill in the Gulf of Mexico [5], [22] and are used for detection and monitoring of harmful algae [23]. However, the vast expanses of the Pacific Ocean have remained a challenge to these systems, despite the clear need to sample remote features 100s to 1000s of kilometers offshore [24], [25].

Wave Gliders are a distinctly different class of AUVs from many previous gliders in that they are wave-propelled with continuous diurnal solar panel support of electrical systems. They are new to the oceanographic community and little published information on their long-term deployments (persistent presence) in the ocean is available. The design of the propulsion system necessarily restricts them to the surface. Thus, sensors deployed directly on the Wave Glider are limited to a two-dimensional exploration of the ocean. However, the mode of propulsion allows tremendous range and duration at the air-sea interface. In November 2011, Liquid Robotics, a private company, launched four wave-powered gliders from San Francisco, CA for a trans-Pacific voyage (the Pac-X crossing). They transited to Hawaii where they were recovered, serviced and deployed to travel in pairs to Australia and Japan. Two arrived in Australia, one in Dec 2012 (the *Papa Mau*) and one in February 2013 (the *Benjamin*), while the two destined for Japan (the *Piccard Maru* and the *Fontaine Maru*) both ran into technical difficulties and did not arrive at their destination. The privately funded and collected data set was then made public by the manufacturer (Liquid Robotics, Inc., Sunnyvale, California).

The dataset acquired during these missions is unique in its continuity and spatial extent by single autonomous vehicles. However, this is not a data set intended to be used for calibrating satellite data or planned to yield new oceanic insights. The effects on glider sensors and the utility of the resulting data stream during such prolonged Wave Glider deployments have not been evaluated. As a first step in exploring the utility of these platforms for long-term deployments, we have examined the data collected by Liquid Robotics for systematic problems, compared it to satellite observations where possible, and identified regions where novel biological observations were possible. The gliders were deployed just a few months after the launch of the Aquarius satellite to measure sea-surface salinity (SSS) [26], thus providing the opportunity to compare the gliders’ *in situ* data to this relatively new satellite data stream. Our evaluation revealed a rich continuous data stream with evidence of both a phytoplankton aggregation event and a low salinity layer in the western Pacific, as well as a number of confounding problems linked to both sensor calibration and location.

## Materials and Methods

### Glider Data

**Figure 1:**
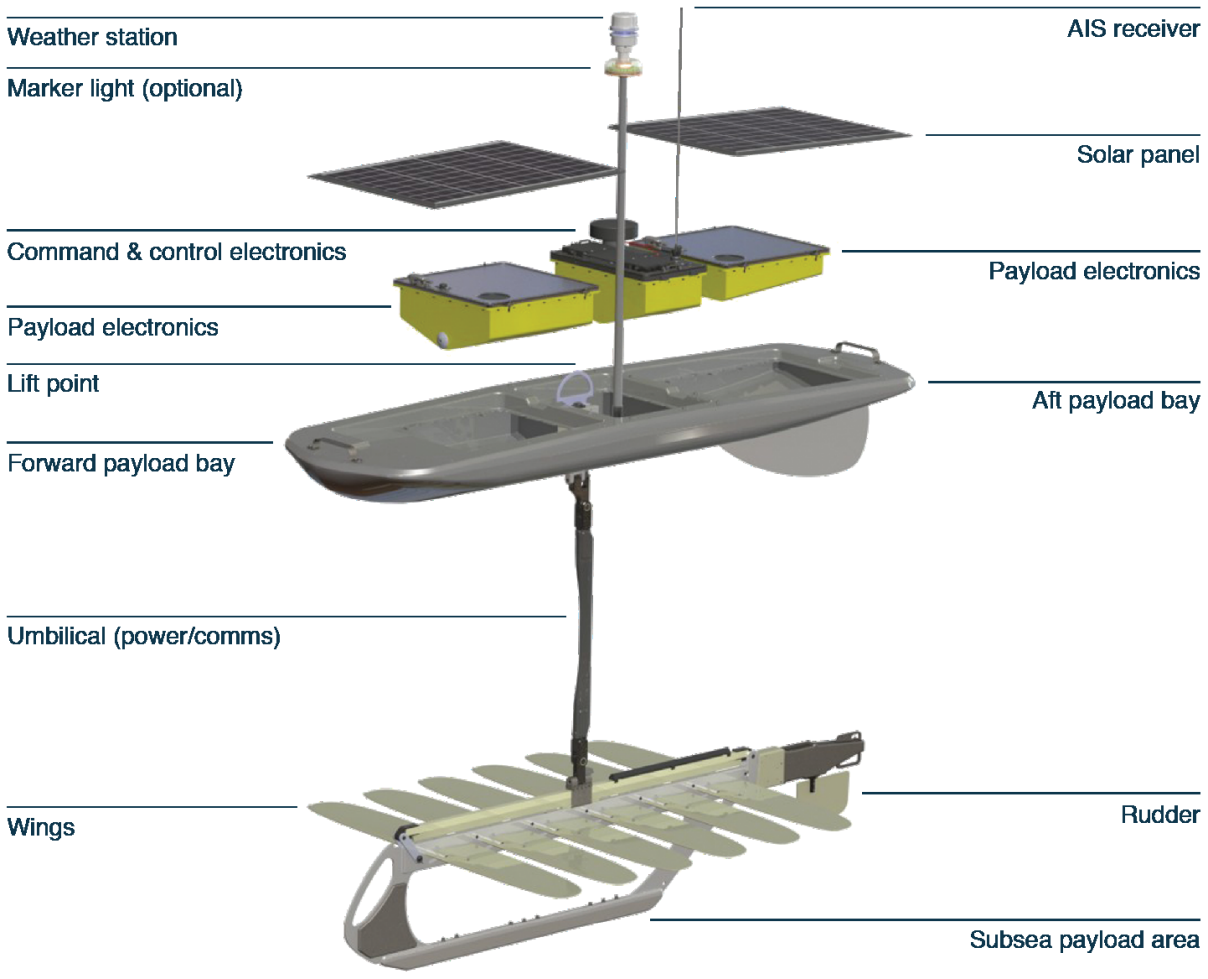
The Wave Glider long-duration autonomous vehicle used in the Pacific crossings. The chlorophyll, oil CDOM, and turbidity sensors were located in the payload bays in the surface float. The CTD sensor was located in the glider subsea payload area.

The Wave Glider (Fig. 1) is an autonomous sampling system that uses a series of wings located on a sub body 7 m below the surface to propel the vehicle forward using the vertical motion induced by wave motion [18]. The surface payload system (termed the float) is equipped with solar cells that provide power for the sampling systems, navigation systems and satellite telemetry links. For the Pac-X crossing (Table 1), each glider supported 4 sampling systems. These were a Seabird Conductivity, Temperature, Depth (CTD) sensor with a dissolved oxygen probe, a Turner Designs C3 fluorometer equipped to measure chlorophyll fluorescence (460 nm ex/696 nm em; reported in fluorescence units), turbidity (850 nm ex./850 em.; units =  NTU; *Nephelometric Turbidity Units*), and colored dissolved organic material (optimized for poly- and monoaromatic hydrocarbons: termed oil CDOM in this paper as per the manufacturer; 325 nm ex/410–600 nm em.), a Datawell MOSE-G directional wave sensor measuring significant wave height, average wave period, peak wave period, and wave direction, and an Airmar PB200 weather station that recorded air temperature, barometric pressure, wind speed, wind gust speed, and wind direction one meter above the deck of thew. The Turner Designs C3 systems and the Seabird CTD and oxygen sensor were all located in the glider payload bay in the float at a nominal depth of ∼0.2 m. The sensor heads pointed down through the bottom of the float payload bays. The sampling interval for the CTD sensors was 10 seconds, for the C3 sensors was 2 minutes and for the weather data was 10 minutes.

**Table 1:** List of launch and recovery times for each glider in the PacX crossing.

Glider	Event	Time (UTC)	Latitude^a^	Longitude
*Papa Maru*	Initial launch outside SF^b^ Bay	11/18/2011 01:15	34.457	–122.834
	Recovery for inspection	12/07/2011 16:45	36.802	–122.136
	Launch after inspection	12/07/2011 18:53	36.779	–122.102
	Recovery north of Maui	03/14/2012 03:57	20.995	–156.300
	Launch from Hawaii	05/08/2012 02:04	19.996	–155.906
	Recovery in Australia	11/20/2012 01:58	–24.116	152.805
*Benjamin*	Initial launch outside SF bay	11/18/2011 01:37	37.458	–122.846
	Recovery for inspection	12/07/2011 19:28	36.778	–122.106
	Launch after inspection	12/08/2011 00:14	36.788	–122.075
	Recovery north in Hawaii	03/15/2012 19:15	19.999	–155.902
	Launch from Hawaii	05/01/2012 02:06	19.935	–155.981
	Recovery in Samoa	10/01/2012 19:01	–13.781	–172.023
	Launch in Samoa	10/04/2012 02:41	–13.780	–172.022
	Recovery in Australia	02/14/2013 22:23	–23.841	152.331
*Piccard Maru*	Initial launch outside SF bay	11/18/2011 00:54	37.449	–122.819
	Recovery for inspection	12/07/2011 20:38	36.769	–122.097
	Launch after inspection	12/07/2011 21:58	36.782	–122.143
	Recovery north of Maui, Hawaii	03/15/2012 20:50	21.996	–155.915
	Launch from Hawaii	05/09/2012 20:08	19.990	–155.892
*Fontaine Maru*	Initial launch outside SF bay	11/18/2011 00:54	37.449	–122.819
	Recovery for inspection	12/07/2011 22:04	36.778	–122.146
	Launch after inspection	12/07/2011 23:16	36.792	–122.062
	Recovery north of Maui	03/09/2012 00:40	19.980	–155.871
	Launch from Hawaii	04/22/2012 20:49	19.857	–155.947
	Rescue in north Pacific	11/15/2012 00:27	21.060	171.352

a Latitude and Longitude are reported as positive for north and east, negative for west and south.

b San Francisco Bay, California.

Data were stored onboard and relayed via satellite link to Liquid Robotics. Additional information about the sensors is available from the Liquid Robotics webpage (http://liquidr.com); a further description of the Wave Glider and its acoustic signature is provided elsewhere [18], [27]. Sensor calibration is a critical issue in missions of such long duration. Pre- and post-calibration data for the 3 gliders recovered was obtained from Liquid Robotics and is listed in Text S1. The Seabird CTD was calibrated by Seabird Electronics (Seattle, WA). Salinity drift ranged from +0.00200 to +0.00210 PSU month^−1^; temperature drift ranged from +0.00001 to 0.00042°C per year. Calibration sheets provided by Seabird are available in Text S1. The Turner C3 sensors were calibrated by Liquid Robotics (chl fluorescence  =  1.0119 mg ml^−1^ Basic Blue 3; crude oil = 250 mg L^−1^ quinine sulfate; turbidity =  3000 NTU turbidity standard) as per Turner Designs recommendation. Range, accuracy and detection limits for the Turner C3 and Seabird gpCTD are presented in Table S1 in Text S1. A summary of instruments and calibration information can be found in Table 2.

**Table 2:** Summary of glider sensors calibration and quality control decisions.

Glider	Measurement	Calibration^1^		Compared	Suspect data
		Pre-	Post-	with satellite^2^	
*Papa Maru*	Temperature	Seabird	Seabird^3^	Y, r^2^ = 0.99	
	Salinity	Seabird	Seabird^3^	Y, r^2^ = 0.79	
	Chl Flu	LR	LR	Y, r^2^ = 0.93^1^	9/2012 onward
	Oil CDOM	LR	LR	N	All daytime
	Turbidity	LR	LR	N	9/2012 onward, possible bubble artifacts
*Benjamin*	Temperature	Seabird	Seabird	Y, r^2^ = 0.99	
	Salinity	Seabird	Seabird	Y, r^2^ = .77	
	Chl Flu	Liquid Robotics	Liquid Robotics	Y, r^2^ = 0.90^1^	Nov-Dec 2011
	Oil CDOM	LR	LR	N	All daytime. Aug 2012, Feb 2013
	Turbidity	LR	LR	N	Nov-Dec 2011, Dec 2013, possible bubble artifacts
*Piccard Maru*	Temperature	Seabird	lost	Y, r^2^ = 0.997	
	Salinity	Seabird	lost	Y, r^2^ = 0.813	
	Chl Flu	LR	lost	Y, r^2^ = 0.172^1^	Nov-Dec 2012, 7/2012 onward
	Oil CDOM	LR	lost	N	All daytime
	Turbidity	LR	lost	N	possible bubble artifacts
*Fontaine Maru*	Temperature	Seabird	Seabird^3^	Y, r^2^ = 0.99	
	Salinity	Seabird	Seabird^3^	Y, r^2^ = 0.86	
	Chl Flu	LR	LR	Y, r^2^ = 0.03^1^	7/2012 onward
	Oil CDOM	LR	LR	N	All daytime
	Turbidity	LR	LR	N	Possible bubble artifacts

LR =  Liquid Robotics, Seabird  =  Seabird Electronics, Y = yes, N = no.

1 Calibration data in Text S1.

2 Compared with VIIRS chlorophyll.

3 calibration drift reports in Supplemental Information.

Nominal detection limits are 0.025 μg L^−1^ for chl, 0.2 ppb for crude oil and 0.05 NTU for turbidity; however, chlorophyll fluorescence was not calibrated directly to extracted chlorophyll. Pre and post calibration runs for the chlorophyll sensors indicated a factor of 4 difference in the slope suggesting a dilution error at Liquid Robotics during the standard preparation. Data is presented only as fluorescence units to reflect the uncertainty in inter-glider comparisons. Incorrect preparation of the turbidity standard precluded accurate pre and post-mission calibration at Liquid Robotics, but a post-mission examination of the sensors by Turner Design indicated no significant change in performance. The temperature data standards used by Seabird are the Gallium Melt Point (GaMP) and Triple Point of Water (TPW) fixed point cells. The Standard Platinum Resistance Thermometer (SPRT) is calibrated in the fixed point cells with associated instrumentation, and then the SBE3 bath temperature references are calibrated against SPRT. The SBE4 bath conductivity references are calibrated in seawater and referenced against IAPSO Standard Seawater using a Guildline AutoSal. We are unaware of any concurrent water sampling taken to validate the Wave Glider sensors during deployment.

Sensor selection and instrument placement on the Wave Gliders was determined by Liquid Robotics. Liquid Robotics planned and executed the recovery, servicing and photodocumentation of the gliders. In this study, we examined a subset of data from the CTD sensors, the C3 sensors and the weather/wave instrumentation. Data were eliminated for times that the gliders were out of the water and when the CTD pressure values (indicative of depth) were negative (i.e. above the surface of the water). Oxygen data was not examined since the near-surface location of the sensor insured maximum ventilation with the atmosphere. As a result, values should be at or near saturation values determined by temperature. Bubble injection at the surface would also lead to supersaturation at times, and the resultant manipulations required to extract useable data were considered too complex for the scope of this paper.

The PacX data presented is unprocessed and unfiltered, with two exceptions. To compare the glider data against satellite data, daily averages were computed for the glider data. An aggregation metric for the western N. Pacific chl bloom was created using the mean fluorescence values (65 fluorescence units) for the period when *Piccard Maru* left Hawaii until its loss in November. The numbers of fluorescence spikes exceeding 3 times this value in a 12-hour period were summed to give a count. During this period, the glider was generally moving between 0.32–0.50 m s^−1^ (0.6–0.9 knots) based on the telemetered navigational information.

Glider data are publically available at http://slab.liquidr.com/fetch/. During the course of this analysis, a revised data set was provided. In this revised data set, Liquid Robotics corrected inconsistencies between different downloadable data files. The exact nature of the inconsistencies was not clarified although additional data was in the revised files. Inspection of plots from the two sources revealed only minor differences. The data used in the paper as well as all calibration data are available at the National Oceanographic Data Center ( http://www.nodc.noaa.gov), accession number 0114435 (http://www.nodc.noaa.gov/cgi-bin/OAS/prd/accession/download/0114435).

### Specific reporting and ethics requirements

During the course of the missions, the gliders entered multiple exclusive economic zones. Liquid Robotics was responsible for determining when and where national exclusive economic zones required permission for sampling (Text S1). The authors had no role in this process. No protected species were sampled. No permits were required for these collections. Although Liquid Robotics provided no details, it is assumed some barnacles and other fouling organisms were killed by exposure to air when the gliders were recovered.

### Satellite Data

The Wave Glider data were compared to satellite data measurements of sea surface temperature (SST) from MODIS (Moderate Resolution Imaging Spectroradiometer), chlorophyll (chl) from both MODIS/Aqua and VIIRS *(Visible Infrared Imager Radiometer Suite*), sea surface salinity (SSS) from the Aquarius mission, sea surface height (SSH) from AVISO (Archiving, Validation and Interpretation of Satellite Oceanographic data) altimetry and Niiler Climatology, and precipitation from the Tropical Rainfall Measuring Mission (TRMM). The MODIS SST and chlorophyll data were at 0.025° (∼2.5 km) resolution, the VIIRS data were at 0.04° (∼4 km) resolution, the SSS data were at 1° (∼100 km) resolution, AVSIO data were at 0.3° (∼30 km) resolution and the rainfall data were at a 0.25° (∼25 km) spatial resolution. Daily images were used for the match-ups with the glider data. Satellite match-ups with the glider data were made by averaging all satellite data within a two-day temporal window and within a spatial radius of 0.1° for SST and chlorophyll, 1° for salinity, and 0.5° for rainfall. Monthly composites of Aquarius SSS, VIIRS chlorophyll, and AVISO SSH were used as overlay maps of the glider tracks except where otherwise noted. Except for the TRMM data, satellite data were obtained from http://coastwatch.pfeg.noaa.gov/erddap/ [28]. The TRMM data were obtained from http://mirador.gsfc.nasa.gov.

The Aquarius SSS and the VIIRS chl data are both relatively new data streams; both were launched just a few months before the Pac-X gliders. The Aquarius/Satélite de Aplicaciones Científicas (SAC)-D satellite is a collaborative effort between NASA and the Argentinian Space Agency Comision Nacional de Actividades Espaciales (CONAE). It was launched in June 2011 and has been providing global maps of sea surface salinity (SSS) since September 2011 [26]. VIIRS was launched in October 2011 on the Suomi National Polar-orbiting Partnership (NPP) satellite, a partnership between NASA and the National Oceanic and Atmospheric Administration (NOAA). Data from VIIRS is available from Jan 2012. The Ocean Biology Processing Group at NASA Goddard Flight Space Center processed both the VIIRS and Aquarius data. The VIIRS data used was still considered an evaluation product, version 2013.0. The Aquarius data was version 2.

Data analysis and presentation used Ocean Data View [29] and IDL (Interactive Data Language, a software product of ITT Visual Information Solutions). Information on Tropical Cyclone Freda was found at the UN Global Disaster Alert and Coordination System (http://www.gdacs.org/) and the Naval Research Laboratory Monterey websites. NOAA weather information on Tropical Cyclone Freda was found through http://www.webcitation.org/6DK6x2euY.

## Results and Discussion

**Figure 2:**
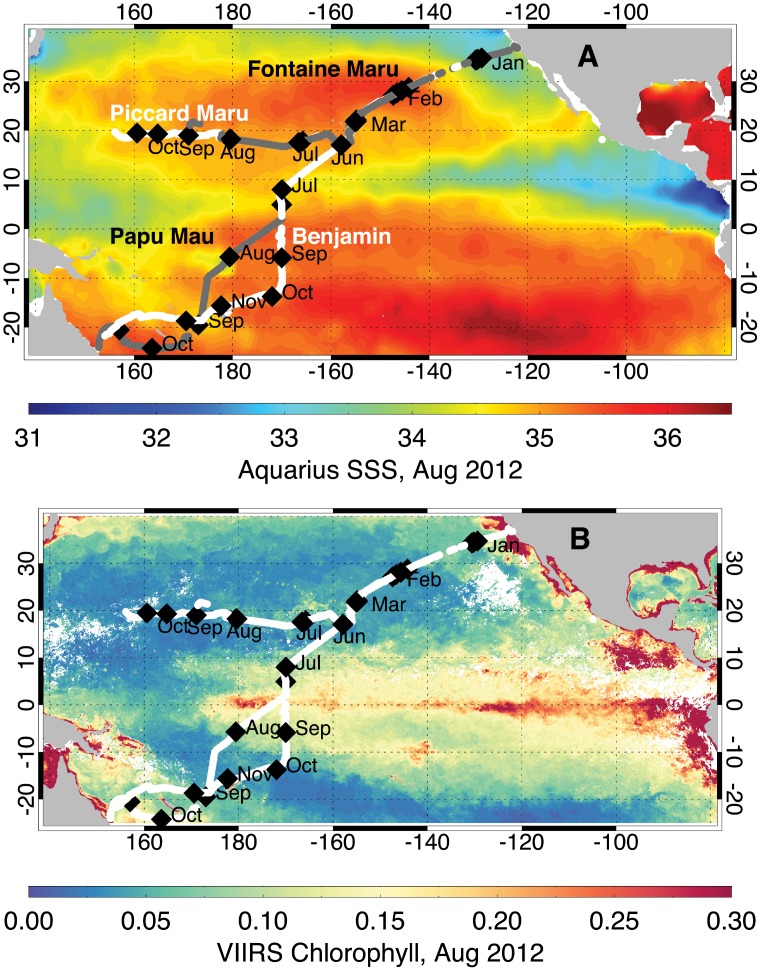
Maps of gliders tracks overlaid on satellite salinity and chlorophyll. The glider tracks are shown overlaid on (a) Aquarius SSS for August 2012 and (b) VIIRS chlorophyll for August 2012. The first day of each month is shown as a black triangle. The glider tracks are labeled in (a) and the tracks of *Benjamin* and *Piccard Maru* are shown in white, and those of *Papa Mau* and *Fontaine Maru* are shown in dark gray. Where the tracks overlap, only the grey tracks can be seen.

All four gliders were launched in San Francisco Bay on Nov. 18, 2011 to begin the trans-Pacific mission (Fig. 2). After an initial test period, they were recovered and inspected in Monterey Bay on Dec. 7, 2011. The gliders then travelled across the N. Pacific to Hawaii, reaching that destination within a few days of Mar. 15, 2012 (Table 1). The gliders were inspected and serviced, and returned to the sea and recovered over several weeks in the vicinity of Hawaii, with the gliders departing for their next destination as follows: *Papa Mau* (Australia; May 8, 2012), *Benjamin* (Australia; May 1, 2012), *Fontaine Maru* (Japan; April 22, 2012), and *Piccard Maru* (Japan; May 9, 2012). The *Papa Mau* was recovered in Australia on Nov. 20, 2012. The *Benjamin* was recovered and re-launched in Samoa (Oct. 1–4, 2012) and reached Australia on Feb. 14, 2013. The *Fontaine Maru* was recovered in the open Pacific by the R.V. *Kilo Moana* (University of Hawaii) on Nov. 25, 2012. Contact was lost with the *Piccard Maru* on Nov. 17, 2012, almost exactly one year after launch. Since that time, periodic contact with the *Piccard Maru* has occurred, but for only 1–2 days approximately at monthly intervals. The glider is disabled and is drifting eastward across the Pacific Ocean (W. Vass, Liquid Robotics). No useful data has been reported to us.

Initial plotting of the data versus time revealed several characteristics of the data sets that were present to some extent in all 4 gliders. During our analysis, selected subsets of the data were deleted due to unresolved quality issues with the data (Table 2). Three of the 4 gliders exhibited serious deviations for some period of time from reasonable environment values with one or more of the C3 sensors during the transit. These deletions and the reasons for them are discussed under the individual sensors below. All four gliders were recovered at least once in Hawaii, inspected and returned to the ocean. Photodocumentation of the sensor heads provided by Liquid Robotics revealed various degrees of fouling by gooseneck barnacles on the C3 and CTD sensor head (Fig. S1–S3). In some cases, the barnacles appeared to be within the sensor field; however, there was no clear relationship seen in the data. The most problematic drift (the final months of the *Papa Mau* C3 chl fluorometer data) could not be resolved since the sea conditions during recovery prevented photodocumentation of the sensors (Luke Beatman, Liquid Robotics, personal communication).

### Glider and satellite comparisons

**Figure 3:**
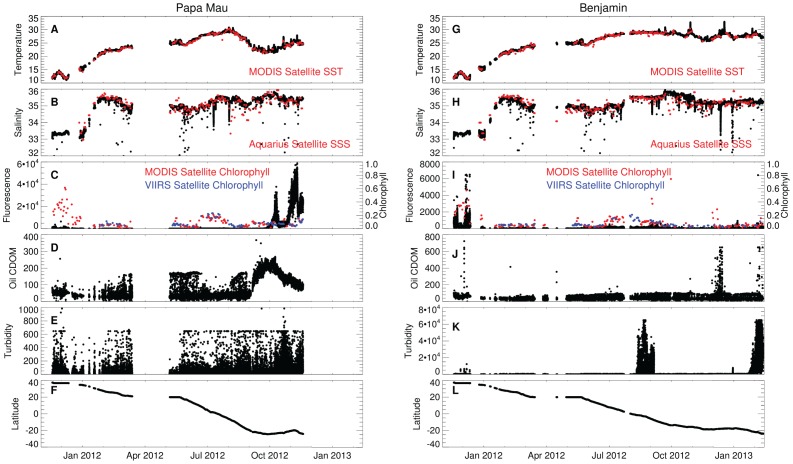
Times-series of CTD and C3 data for *Papa Mau* and *Benjamin*. Time-series for the complete mission of (a & g) temperature, (b & h) salinity, (c & i) fluorescence, (d & j) Oil CDOM, (e & k) turbidity and (f & l) latitude for the *Papa Mau* and *Benjamin* gliders. Synoptic temperature values from the MODIS satellite are overlaid on a & g in red. Synoptic salinity values from the Aquarius satellite are overlaid on b & h in red. Synoptic chlorophyll values from the MODIS satellite are overlaid on c & i in red, which are on a different scale than the glider fluorescence data. Note that the scales for (c & i) chlorophyll fluorescence (d & j) Oil CDOM and (e & k) Turbidity are different for the two gliders.

**Figure 4:**
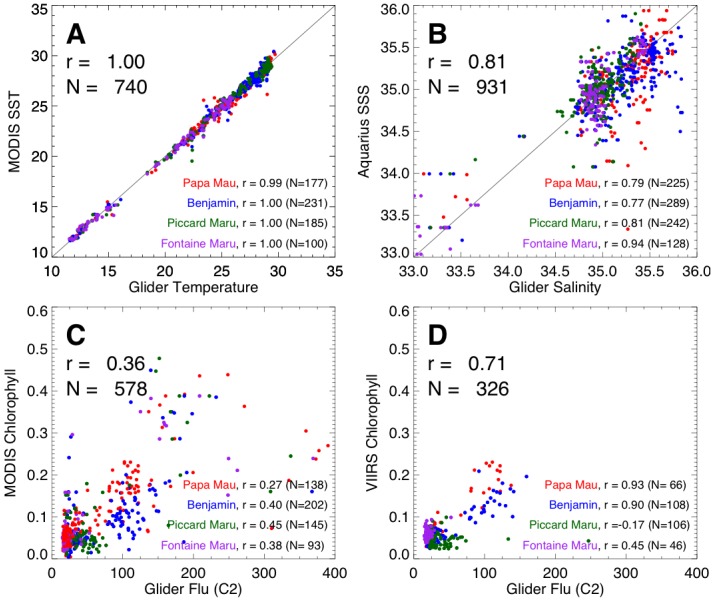
Correlations between glider and satellite data. Correlations between (a) daily averaged glider temperature and MODIS SST, (b) daily averaged glider salinity and Aquarius SSS, (c) daily averaged glider fluorescence and MODIS chlorophyll, and (d) daily averaged glider temperature and VIIRS chlorophyll. Points are color-coded by glider; *Papa Mau* is red, *Benjamin* is blue, *Piccard Maru* is green and *Fontaine Maru* is purple. The correlation for all glidersis shown, as well as the correlations for each glider individually.

On missions of this duration, assessing instrument integrity and data validation are critical to determine the utility of the collected data. The complete time-series of data from the *Papa Mau* and the *Benjamin* are shown in Fig. 3. The data is unfiltered and unsmoothed. The complete data for the *Piccard Maru* and *Fontaine Maru* are presented in supplemental materials (Fig. S4, S5). All gliders have gaps in the data at various points of the mission. The data gap from mid-March-May 2012 occurred during recovery in Hawaii (Table 1). Significant gaps also exist in the N. Pacific crossing prior to this due to weather limiting the solar cell charging. As a first pass for data quality, satellite values of sea surface temperature (SST), sea surface salinity (SSS) and chlorophyll (chl) measured along the track were overlain on the respective glider data (Fig. 3a, b, g, h). Data from all four gliders aligned well for temperature and showed no evidence of systematic differences. The satellite and glider values for temperature and salinity are well-correlated, with r values of 1.0 and 0.81 respectively (Fig. 4). The lower salinity correlation reflects a sampling distinction between the satellite and glider. The Aquarius microwave sensor sample sonly the top 1 cm of the ocean [30] whereas the glider CTD system is 0.2 m below the surface. In most oceanic circumstances, this difference is inconsequential. However, as we note below, in regions of heavy rainfall (the intertropical convergence zone; ITCZ and the South Pacific Convergence Zone; SPCZ) the historical data indicates salinity gradients can exist over this depth range [31]. For example, the two gliders which crossed these regions, the *Papa Mau* and the *Benjamin*, have lower salinity correlation values (r  =  0.79 and 0.77) than the two gliders (the *Piccard Maru* (r = 0.81) and the *Fontaine Maru* (r = 0.94)) which did not. Thus, the lower salinity correlation can be attributed to both the systematic differences in the satellite versus glider sampling as well as the inherently lower accuracy (± 0.2 PSU, [26]) of the Aquarius system versus the Seabird CTD.

The Turner C3 data for chl fluorescence, oil CDOM and turbidity is more challenging to interpret. In the initial inspection of the data, it was apparent that the *Papa Mau* chl fluorescence data after Oct. 2012 contained a serious artifact (Fig. 3c; note the scale differences between Fig. 3i) when compared to the *Benjamin* data from approximately the same region. The *Papa Mau* fluorescence values increased by two orders of magnitude, from ∼300 fluorescence units to > 20,000 in October 2012, declined to 3000–4000, and then increased again to > 50,000 just before recovery (Fig. 3c). Satellite ocean color chl revealed no evidence of a chlorophyll increase of that magnitude, nor are these values consistent with comparisons to the richer waters of the California Current. A nearly coincident drift in the oil CDOM data occurred as well with values 3–4 times that noted in the California Current. The direction of the drift is opposite (the oil CDOM maximum occurred well before the chl fluorescence maximum and was declining), so a single source seems unlikely. Since there was no photodocumention of the sensor head upon recovery, this data is considered problematic and was excluded from analysis. Similar deviations are seen in the *Benjamin* data from the turbidity sensor in August 2012 and late Jan. 2013. During these periods, there was great variability (10,000s of NTU) in the turbidity signal and values rarely dropped to the background of 40–50 NTU. A similar drift after Hawaii was evident in the *Fontaine Maru* data (Fig. S5) shortly before loss of the glider. We interpret these periods to be intervals when some object was in the sensor window, possibly the fouling organisms seen in the photodocumentation (Fig. S1–S3). Data in these intervals were discarded from further analysis. A feature in the *Benjamin* oil CDOM data from ∼10–15 Dec. was difficult to interpret. It was within a reasonable range for the data (<700 AU) and background values of 20–27 were observed throughout the spiking to higher values. Caution was required in evaluating these features. Intense spiking between background values occurred in all of the various gliders’ C3 data at some point. These patterns could only be resolved by expanding the scale to look at daily data. An additional curiosity in the data was the apparent truncation of all three fluorescence channels at a nearly constant value sporadically, thus creating the effect of a line through the data. After discussions with the vendor (Turner Designs), this was determined to be the consequence of the autogain feature of the C3 having inadequate time to reset the gain to keep the value on scale. Multiple flashes of the excitation signal are required to properly set the gain, and values collected before this are registered as the maximum on-scale value permitted by the range. Thus, off scale data appear as a line through the data equal to the maximum permissible value at a gain setting. This was not considered a serious problem, although the absolute magnitudes of these particular spikes were invalid. This problem is treated in vertical profiles of oceanographic data by bin averaging the data into 1-meter increments (∼ 1 minute of real time) from its multiple samples per second resolution. The presence of a pronounced diel cycle in chl fluorescence made this somewhat problematic, and the data was left unfiltered to preserve its overall sense.

After removal of problematic data, regressions on the chlorophyll fluorescence data were made against two satellite chl products: MODIS and VIIRS. MODIS is at the end of its operational life; VIIRS was launched recently with data available from Jan 2012 on. These regressions suffer from a problem inherent in the low dynamic range of chlorophyll along this transect (see Figure 2a). The highest chlorophyll values occurred in the coastal California Current, a region of significant horizontal variability due to the complex fronts that occur in the upwelling zone. This creates a challenging comparison between daily averages of glider chl fluorescence at meter scales to the intermittent satellite measurements at kilometer scales. Because the VIIRS dataset starts in Jan 2012, after the gliders were out of the California Current, the VIIRS regressions do not have data from this highly variable region, leading to a better correlation with VIIRS than with MODIS. Only two gliders crossed the other area of significant chlorophyll, the equatorial Pacific. *Papa Mau* and *Benjamin*, the gliders that traversed the equatorial bloom area, had a good correlation with VIIRS chl (r =  0.93 and 0.90). All other correlations were poor, with values < 0.5. Inspection of the combined data plots shows that there was a 2–10 fold range in chl fluorescence associated with any particular MODIS or VIIRS chlorophyll value. Individual gliders had different fluorescence values for the chlorophyll minimum seen in the open Pacific.

### Turbidity data

**Figure 5:**
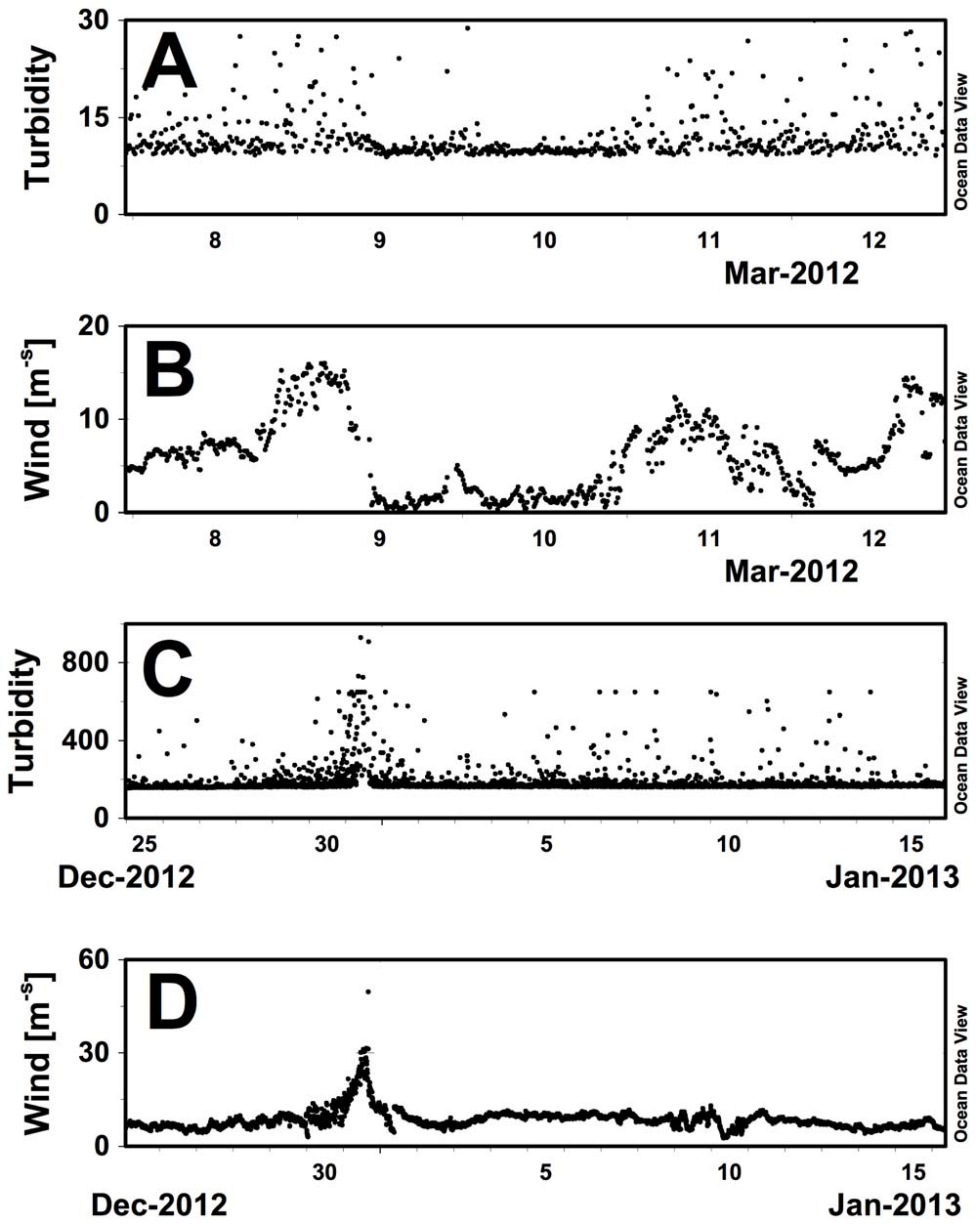
Time-series of turbidity data illustrating the effect of wind speed on readings. (a) Turbidity, (b) Wind speed, *Benjamin* 10 March 2012 location was 20.016 °N 155.942 °W; (c) turbidity, (d) Wind speed, *Benjamin* 31 Dec. 2012 location was 17.947 °S 161.398 °E. The high wind event was Tropical Cyclone Freda. Note the scale change in wind and turbidity between Fig 5a, b and Fig. 5c, d.

Our initial concept for this data set was to use spiking in the chl fluorescence and turbidity data as a proxy for large particulate aggregates [21], [32], [33]. The turbidity data contains a significant amount of spiking suggestive of large particles; however, this particular sensor responses to bubbles as well (pers. comm Turner Designs). When daily averaged turbidity was regressed against daily wind speed, the correlation was poor, suggesting no impact (data not shown). However, examination of the data to compare zero wind events (dead flat calm) to hurricane force winds (Tropical Cyclone Freda) revealed that variability in the turbidity disappeared during calm conditions (Fig. 5a, b), and reached a maximum during the tropical cyclone (Fig. 5c, d). In T.C. Freda, the baseline completely disappeared into the spiking (Fig. 5c) indicating a significant change in the base state. While only one tropical storm was encountered, the pattern during calm conditions was repeated several times. In each case, the turbidity variability decreased to almost monotonic values for the extent of the calm condition. Minimum C3 turbidity values probably represents an accurate measure. However, any deviation from this cannot be distinguished from bubbles. By its design, the Wave Glider is propelled at the surface like a surfboard and bubbles of all sizes will roll along the bottom of the float. Microbubbles are of particular concern since they will not rapidly ascend and are likely the source of much of the noise. No further analysis of the turbidity data was attempted.

### Diel signals

**Figure 6:**
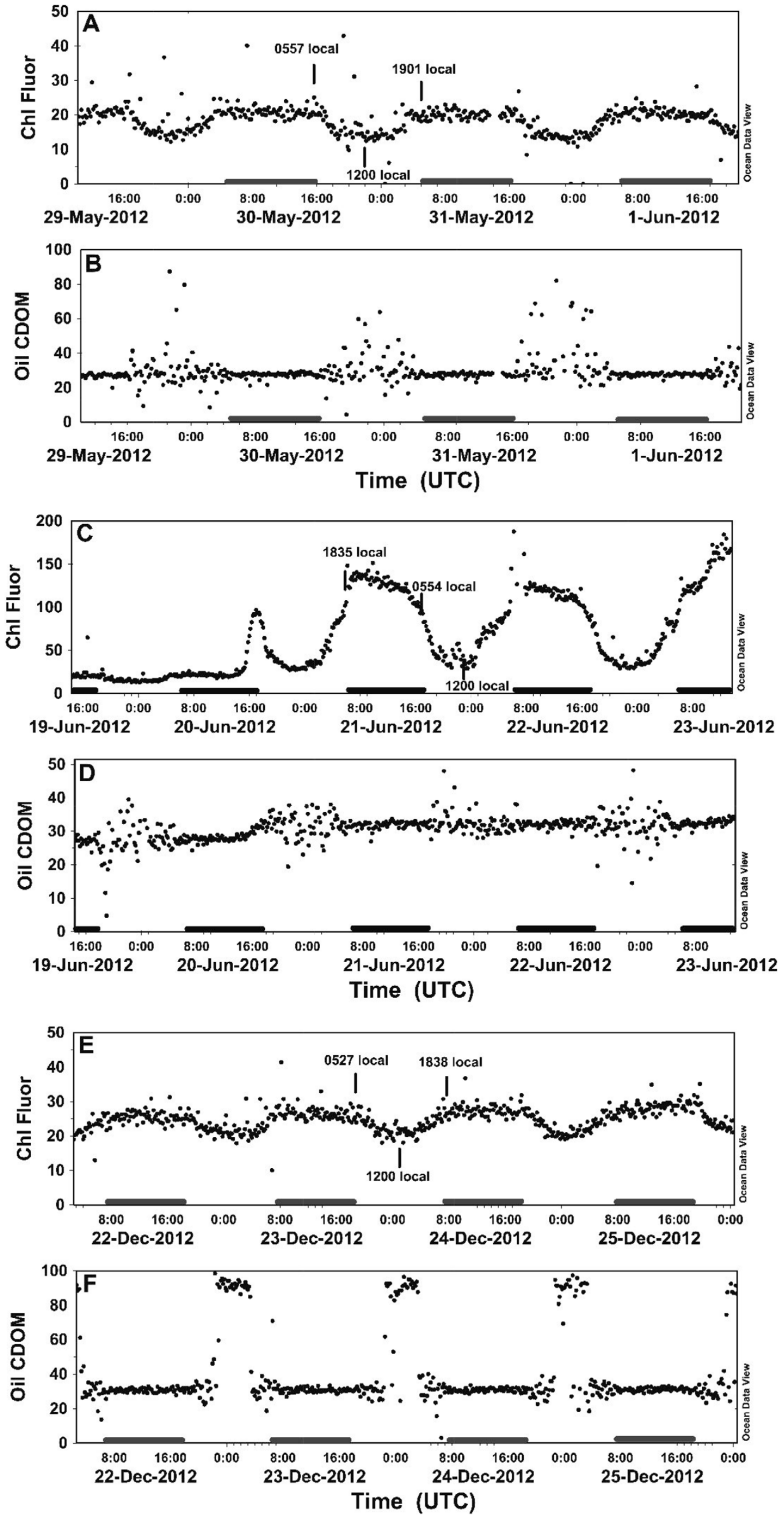
Diel patterns in chlorophyll fluorescence and oil CDOM in the major geographic regions sampled. Time axes are in UTC. Shaded areas are local nighttime. Vertical lines are local dawn, noon and sunset. Diel patterns in chlorophyll fluorescence (a,c,d) and oil CDOM (b,d,e). Panels (a, b.) *Papa Mau* off Hawaii (17.056 ° N 157.796 ° W) (c, d). *Papa Mau* in the Equatorial Pacific (9.466 ° N 168.13 ° W). (e) *Benjamin* off New Caledonia (17.565 ° S 164.387 ° E).

A clear diel signal (Fig. 6) was present in the C3 chl fluorescence and oil CDOM data for almost the entire sampling period and was noted in data from California to Australia. During the significant fluorescence excursions noted earlier, this physiological cycle of the phytoplankton completely disappeared. The chl pattern was a diurnal minimum and nocturnal maximum and was seen even in the highly variable chlorophyll distribution off California. The entry into the equatorial Pacific was evident as a rapid increase in chlorophyll fluorescence on 20 June 2012 late in the scotoperiod (Fig. 6c,d). In the low biomass waters of the North and South Pacific Ocean, the diel fluorescence range was ∼2 fold (fig. 6a, 6e). The equatorial Pacific diel chl pattern was more extreme with 5–6 fold variation between the nocturnal maximum and diurnal minimum (Fig. 6c). In this latter zone of elevated biomass, the nocturnal chl fluorescence maximum peaked and declined well before sunrise, with the fluorescence decline beginning about 4 hours prior to sunrise (Fig. 6c,d, Fig S7). In contrast, the more oligotrophic waters north and south of the regions reached a monotonic plateau for most of the night and declined at or after sunrise (Fig. 6a,e).

A diel cycle was present in the oil CDOM data as well. The exceptions were the periods when baseline drift was extreme (Fig. 3d) suggestive of either sensor failure or the presence of a large fouling signal. The diurnal maximum was in the form of increased positive scatter, or in many cases, a significant positive deflection of the oil CDOM baseline. We considered photochemical degradation of CDOM into highly fluorescent compounds; however, discussions with the vendor (Turner Designs) indicated that the optional light shield was not installed on these C3 fluorometers. Rather, a custom copper plate was installed to reduce fouling and this precluded installation of the light shield (Fig. S1–S3). The light shield is specifically designed to eliminate solar excitation of the target molecules. Thus, we concluded that the recurrent diel signature was associated with solar-induced stimulation of the oil CDOM component and that it would be impossible to separate out any photodegradation events. Other than a gradient out of San Francisco Bay, there were no important gradients in the oil CDOM data, and this data set was not examined further.

### Tropical Cyclone Freda

**Figure 7:**
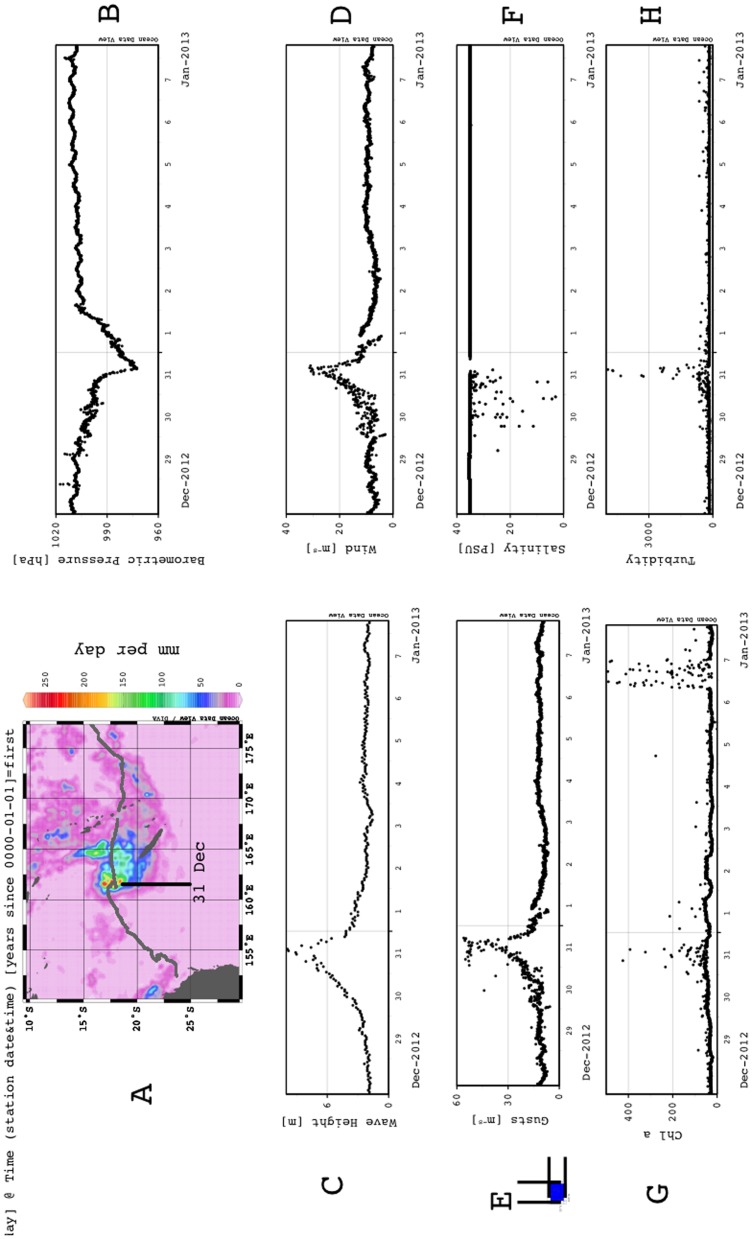
Tropical Storm Freda. (a) Glider track overlaid on TRMM precipitation estimate for 31 Dec. 2012. (b) Barometric pressure. (c) Significant wave height. (d) Average wind speed. (e) Maximum wind speed. (f) Salinity. (g) Chlorophyll a fluorescence. (h) Turbidity.

Storms are one of the hazards of research operations in the open sea. Shipboard operations have adaptive options to leave the area or modify operations and weather the storm. Slow-moving autonomous vehicles have fewer options, or may intend to sample the storm. Thus, it was of great interest when we noted that the track for *Benjamin* intersected with the path of Tropical Cyclone Freda on 31 Dec. 2012 (Fig S6). Tropical Storm Freda formed near the Solomon Islands on Dec. 27, 2012 and moved southwest towards New Caledonia. RSMC Nadi (http://www.met.gov.fj/) classified it as a category 4 severe tropical cyclone on 30 Dec. 2012 with maximum winds of 115 mph (185 km h-^1^). After this peak, the storm weakened due to wind shear as it moved to the southeast and was downgraded to a category 2 tropical cyclone on 1 January 2013, and later that day, RSMC Nadi downgraded it further to a tropical depression. Minimum recorded pressure was 940 hpa with maximum rainfall accumulation of 496 mm (UN Global Disaster Alert and Coordination System). A tracking map for *Benjamin* and TC Freda (Fig. S6) shows the intersection in the Coral Sea (eye position at 16.4°S 161.1°E ± 30 nautical miles on 31 Dec. 2011 0000Z; Fig. 7). *Benjamin*’s sensor packages operated during the storm (Fig 7b-7h) and recorded maximum winds of 56.2 m^−s^ (104 knots; Fig. 7e), significant wave heights of 9.9 m (Fig. 7c), and notable increases in turbidity (storm-induced bubbles, Fig. 7h) and decreases in salinity (Fig. 7f) associated with the storms precipitation and/or bubbles (Fig 7a). *Benjamin*’s onboard meteorological package recorded a low pressure of 972.5 hPa, well above with the lowest pressure (940 hPa) reported, but it was ∼40 km from the eyewall. The Seabird CTD pressure sensor also recorded the decrease in atmospheric pressure, reporting an apparent depth of approximately –0.4 m. NOAA weather reported maximum winds on 31 Dec 2012 as 105 knots (54 m s^−1^) with gusts to 130 knots (66.9 m s^−1^).

### Hydrographic data

**Figure 8:**
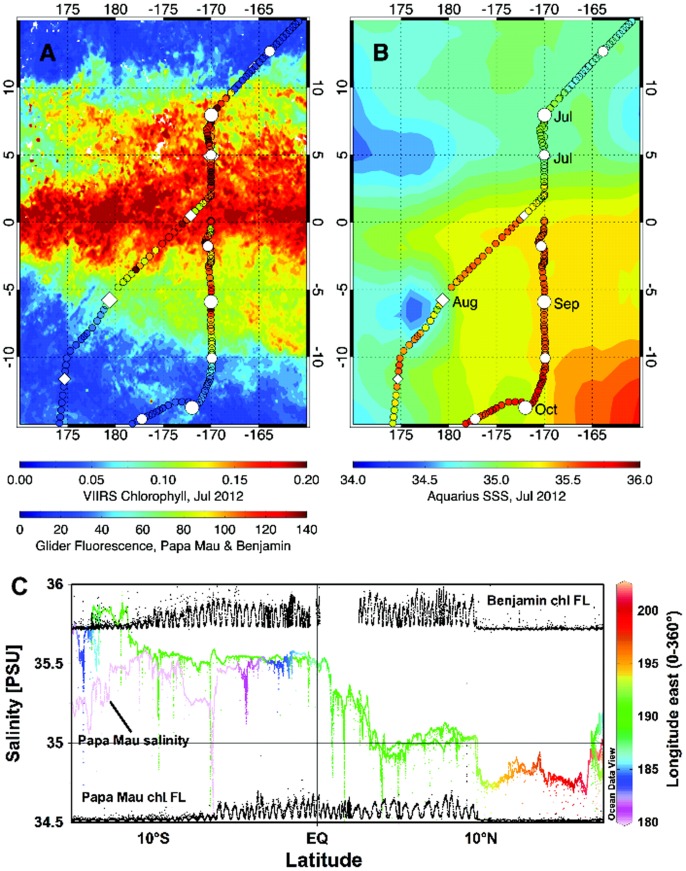
Glider tracks overlaid on satellite chlorophyll and salinity for the equatorial crossings. The glider tracks for *Papa Maru* and *Benjamin* are shown overlaid on (a) VIIRS chlorophyll for July 2012 and (b) Aquarius SSS for July 2012. The glider tracks are color coded by the value for glider fluorescence and salinity. The first day of each month is shown as a large white diamond, the fifteenth of the month is shown as a smaller white diamond. *Benjamin* followed a path along 170°W, while the *Papa Mura* took a more western route. (c). chl fluorescence (relative units) overlaid on salinity tracks as a function latitude. Longitude is color-coded for the salinity tracks for reference to panel (b). The *Papa Mau* salinity track is labeled, the *Benjamin’s* salinity track overlays this until ∼1°S and is not labeled due to space constraints.

The gliders sampled the major oceanographic features of the Pacific Ocean. The California Current and Equatorial Pacific with their expected biomass changes (Fig. 3a, b, S4, S5) were the dominant features. The high biomass, low salinity waters of the California Current are evident in the initial two months of deployment as low salinity, moderate chlorophyll fluorescence areas (Fig. 2b, 3). The upwelling zone within it is visible as the high chlorophyll values along the US west coast (Fig. 2b). All four Wave Gliders transited along the same path to Hawaii. The California Current is a broad feature, about 800 km wide [34] and is evidenced in the gradual increase of salinity as the platforms entered the N. Pacific gyre (Fig. 2a, 3, S8). The North Pacific subtropical front is evident in the data as a salinity increase from approximately 34.8 to 35.2 near 32°N (Fig S8). All 4 gliders were closely spaced along a single track at this time, and no longitudinal coverage along the front was available. The north STF extends across the entire Pacific Ocean, curves southward and the eastern boundary, where it separates the fresher water of the California Current from the saltier water of the North Pacific Gyre [35]. Another salinity front (south subtropical front) was evident just north of Hawaii as the gliders left the high salinity waters of the gyre (Fig. S8) and entered the wintertime subtropical current [36]. In neither case was a change in the phytoplankton biomass (chl fluorescence) evident. The other feature of interest is the equatorial region. Only two of the gliders, the *Papa Mau* and the *Benjamin*, crossed the equator. They transected the equatorial chlorophyll bloom (Fig. 2b, 8a, c) and then the region of lower salinity along 10°N beneath the ITCZ (Fig. 2a, 8b,c).

### Equatorial Pacific

**Figure 9:**
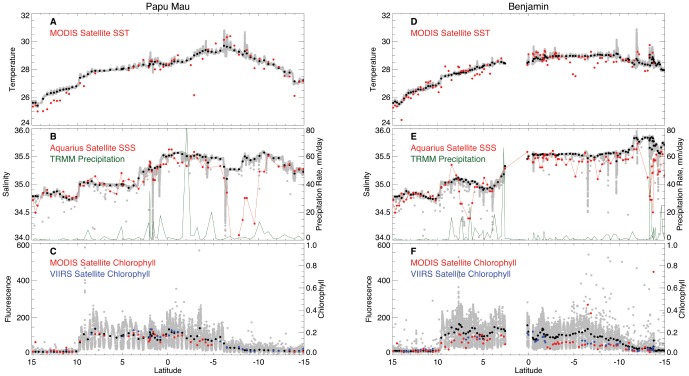
Time-series of *Benjamin and Papa Mau* data for the Equatorial crossing. Plots of (a) temperature, (b) salinity and (c) fluorescence for *Benjamin* and (e) temperature, (f) salinity and (g) fluorescence for *Papa Mau* against latitude The glider data at full temporal resolution are plotted in gray, the daily averaged values are in black, and the satellite data are colored. TRMM precipitation data is shown in green on the salinity plots. Both MODIS (red) and VIIRS (blue) chlorophyll data are shown on the fluorescence plots.

The most variable region that the gliders sampled was the equatorial Pacific region (Fig. 2a,b), where upwelling generates higher chlorophyll, and the presence of the ITCZ along 10°N leads to lower salinities (Fig. 2a). Data along the tracks of the two gliders that transected this region (the *Benjamin* and the *Papa Mau*) are shown in Fig. 8a-c. The fluorescence data from the gliders, when scaled to 0–140 fluorescence units matches up well with the satellite chl data scaled from 0–0.2 mg/m^3^, which is consistent with the correlation analysis presented earlier for these two gliders (a 4). The biological response to the equatorial upwelling is clearly evident in the increased glider fluorescence found between 10° N and 5–10 °S (Fig. 8c, Fig. 9). The match-up between the glider and satellite salinity data is better north of the equator. South of the equator, the glider salinity is saltier than the satellite salinities (Fig. 8b). However, an important caveat with overlaying track values on the satellite data is that there can be significant variations in the temporal match ups between the two datasets. For example, the satellite images are monthly composite for July 2012, while the glider data shown was collected between early June through mid October 2012 (Fig. 8, 9).

**Figure 10:**
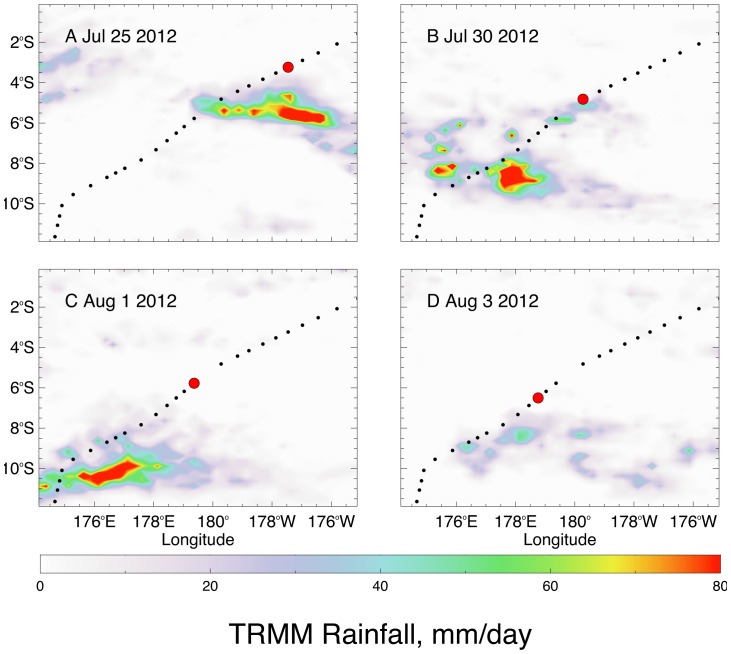
Daily maps of precipitation near the *Papa Mau* tracks south of the equator. Precipitation maps for (a) July 25, (b) July 30, (c) Aug 1 and (d) Aug 3 with daily positions shown of the *Papa Maru* glider. The position synoptic with the precipitation is shown as a large red circle.

The difference between glider and Aquarius salinity data is not due entirely to the temporal differences between the data as can be seen in Fig. 8 and 9, where the glider and satellite data are plotted against latitude. There are several areas of significant discrepancy between the Aquarius and glider salinities. The Aquarius data is fresher than the *Benjamin* data by ∼0.5 salinity between 6°–8°N (Fig. 9b), a zone characterized by lower salinity due to its location under the ITCZ. However it is interesting to note that the *Papa Mau*, which followed the same track but preceded the *Benjamin* by two weeks (Fig. 9b) did not show much of a discrepancy, ∼0.1 salinity at the most. The *Benjamin* transected this region during a period of much heavier rainfall than the *Papa Mau* (Fig. 9b,e). The glider’s CTD sensor is located at a depth of 0.2 m on the glider, and the satellite measurement of salinity is made by a microwave radiometer (top 1 cm) We interpret this to indicate a surface lower salinity water lens less than 20 cm thick. *Benjamin* also sampled another low salinity lens associated with heavy rainfall between 13–14°S (Fig. 9b). The biggest difference between the Aquarius and glider salinities occurred when the *Papa Mau* transected the SPCZ, between 5–10°S and 175°–180°E. While the bolus of Aquarius salinities < 35, and > 1 salinity fresher than the *Papa Mau* salinities appears anomalous (Fig. 8a, 9e), this is part of the band of lower SSS that extends down from the western “fresh pool” created by the heavy rainfall of the SPCZ [37], [38]. While there was no significant rainfall in this area during the *Papa Mau’s* transect (Fig. 9b), significant rainfall in this region preceded the glider (Fig. 10). A simple mixing calculation indicate that 50 mm of rain, well within the observed precipitation, mixed over the top 2 meters of water with salinity  = 35 would decrease the surface salinity to 34.1, thus it is likely that the low salinity signature could extend deeper than just the upper 20 cm in other regions. Surface data (5.5 m) from an Argo float (5902229_124) 140 km northwest (5.5°S, 178.2°E on Aug 3, 2012) from *Papa Mau* during the crossing of this “fresh pool” indicated S  =  35.4, consistent with the glider values (Figure 9e).

The salinity fronts found in this region (Fig. 8c) bounded the biological zonation of the central equatorial Pacific. Both gliders encountered a salinity front at 10° N where a sharp increase from a salinity of 34.25 to 35.1 defined the northern boundary of the higher salinity, westerly flowing equatorial current system and the southern influence of the ITCZ. Chl fluorescence increased 3–5 fold and was maintained as the gliders progressed south through another salinity gradient to 35.5. The *Papa Mau*, on its more westerly path (Fig. 8a), left this region and entered the lower salinity water of the SPCZ (35–35.25 and declining: Fig. 8c) typical of the western Pacific warm pool with a subsequent decline in fluorescence. The *Benjamin* continued south, staying in the higher salinity water mass, until chl fluorescence declined as it entered the higher salinity water of the south Pacific gyre (salinity  =  35.75). *Benjamin* then proceeded southwest towards Australia.

### Open ocean chlorophyll bloom

**Figure 11:**
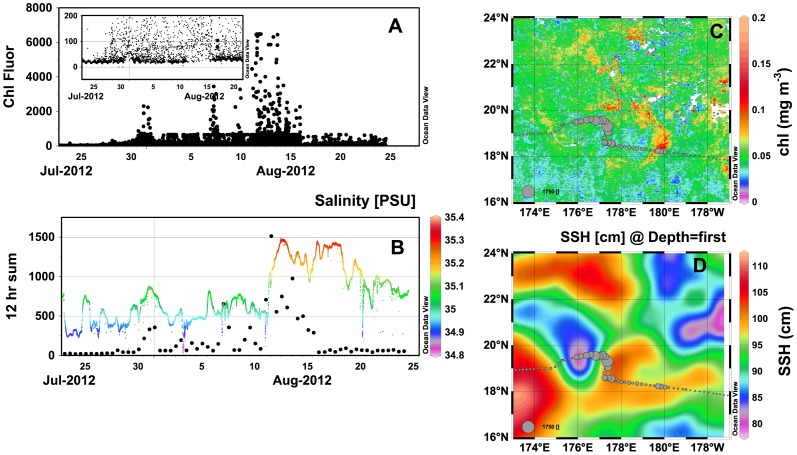
Open ocean chlorophyll bloom sampled by the *Piccard Maru*. (a) Glider chlorophyll fluorescence from the C3 sensor. Inset: expanded view showing diel cycle of chlorophyll fluorescence throughout the event and stable baseline. (b) 12 hour spiking sum and salinity field during the event. Salinity is color-coded by scale on the left. (c) Bubble plot of 12 hr spiking sum overlaid on MODIS chlorophyll distribution (8-day integration composite centered on 5 Aug. 2012). (d) Bubble plot of 12 hr spiking sum overlaid on sea surface height (30 day composite; 1 Aug 2012) from satellite altimetry.

Both the *Fontaine Maru* and the *Piccard Maru* entered an area of the oligotrophic western Pacific in late July 2012 that showed elevated chlorophyll levels in the satellite data (Fig. 11). These areas are of particular interest since they frequently co-occur with blooms of nitrogen-fixing diatom symbioses (*Hemiaulus hauckii* and its symbiont *Richelia intracellularis*) [39], [40].). While satellite chlorophyll can indicate the presence of phytoplankton biomass, diatom symbioses can reach bloom levels without an obvious satellite chl signal [41]. In this latter bloom, the diatom symbioses aggregated into visible macroscopic flocs [41] that are important contributors to summer carbon flux into the deep sea [42]. Aggregation is often transient and difficult to detect; however, optical signals (spikes) in either transmissometer or fluorometer profiles have been shown to be useful in detecting aggregates and quantifying their role in vertical transport [21], [32], [33], [43]–[45]. In the case of open ocean chl blooms in the Pacific, spiking in vertical CTD profiles coincided with diver-observations of large diatom aggregates [46]. There is also evidence that eddy-eddy interactions in the open sea can focus large particulates [47], and in the case of photosynthetic particles, this will appear as fluorescent spikes as well. The fortuitous sampling by these gliders provided an opportunity to examine if the surface fluorescence could be used to document these phytoplankton events via changes in the fluorescence spiking.

The diel range of background chl fluorescence in this region prior to the bloom ranged from ∼10–30 fluorescence units for the *Fontaine Maru* and ∼10–35 fluorescence units for the *Piccard Maru*. On 23 July 2012, the *Fontaine Maru* began a long-term drift in the chl a fluorescence data, eventually reaching chl fluorescence values of 1150 fluorescence units on 11 Aug. 2012 (Fig. S7). This was equivalent to values in the California Current and well outside the range of credible surface values for the open Pacific Ocean. More importantly, the diel pattern in chl fluorescence seen across the Pacific Ocean was completely lost, suggesting the baseline values did not represent a signature from phytoplankton. The glider then went silent for several months, briefly restored contact in mid November, and was recovered by the R.V. *Kilo Moana*.

Although the *Fontaine Maru’s* chl fluorescence conveniently aligned with features of interest in the satellite data, the problematic nature of *Fontaine Maru* data could not support further analysis. Data from the *Piccard Maru* also looked questionable when viewed as part of the total data set (Fig. S6); however, closer examination of it indicated substantial differences from the *Fontaine Maru*’s data. Unlike the *Fontaine Maru*, the *Piccard Maru*’s data did not show an upward drift of the baseline (Fig 11a). While the instantaneous values showed considerably variability, the baseline was steady at ∼10–20 fluorescence units (Fig. 11a). The diel pattern in fluorescence was visible through the area of interest (Fig. 11a inset). We concluded that the fluorescence data reflected processes occurring in the water and supported analysis. There was an increase to 50 fluorescence units from 31 July to 1 August 2012 that coincided with passage through a chl filament (Fig. 11a-c). Beginning on ∼28 July 2012, the variability (spikiness) of the fluorescence data increased dramatically as well (Fig. 11b), and it was this high density of spiking that gave the appearance of a general increase in fluorescence in Fig. S6. We applied an aggregation metric (see Methods) intended to provide quantitative value of the number of spikes per 24 hour that exceeded the mean fluorescence values as well as the diel pattern of chlorophyll fluorescence. The spiking density decreased abruptly on 16 August (Fig. 11a), but in general was more elevated for the duration of the mission west of this area relative to east of the bloom (Fig. 11a). The chl fluorescence was approximately twice background for a period of 14 Aug. to 23 Sept 2012, then returned to pre-increase background.

When overlaid on sea surface height data from satellite radar altimetry (Fig. 11d), the spiking was associated with the region between an anticyclonic and cyclonic mesoscale eddy, a region also associated with a salinity increase of 0.3 suggestive of a front (Fig. 11b). The spiking occurred at the interface of a clockwise flow around an anticyclonic feature (high relative SSH) and a counter-clockwise flow around a cyclonic feature (low relative SSH). The influence of the currents in this interaction zone can be seen in the significant northern displacement of the glider’s trajectory in this region as well (Fig. 11c, d) and the southerly displacement as the glider exited the cyclonic feature at ∼175.5 °W.

### Evaluation

The PacX missions highlighted a number of positive aspects of the Wave Glider long duration flights as well as some of the potential pitfalls. The continuity of data combined with the programmable navigation allowed considerable replication of tracks during the California to Hawaii leg. The divergence of the *Benjamin* and *Papa Mau* after the equatorial crossing captured details of the low salinity lens of the western South Pacific. The durability during Tropical Cyclone Freda was remarkable (Liquid Robotics claims 10 hurricanes, typhoons and cyclones transited by these systems: http://www.liquidr.com/news_events/newsletters/making-waves_1302.html), and suggests a future role for these systems in understanding the development of tropical systems such as the program being developed by the Atlantic Oceanographic and Meteorological Laboratory (NOAA).

The duration of these missions required considerable data validation and quality control. The glider temperature and salinity were well correlated with their respective satellite sensors. Long-term stability and fouling of these sensors was not a serious problem. There are several layers of complexity associated with comparing chlorophyll fluorescence to the ocean color satellite data. A pronounced diel rhythm in chlorophyll fluorescence is well documented [48]–[50] and is linked to biological processes that regulate electron flow in photosystem II [51], [52] in accordance with physiological state and diel patterns of solar radiation. Satellite chlorophyll data is based on wavelength specific absorption by particulates after substantial corrections for atmospheric interference. Thus, they are inherently different measurements of the same property (chlorophyll a). This is compounded by the surface location of the C3 sensor (maximum solar effects) when compared to the satellite chlorophyll measurement over one optical depth (∼30 m for most waters in this study). The chlorophyll fluorescence will also respond to individual large particles common in these waters at the surface such as *Trichodesmium*, colonial radiolarians and various diatom aggregates. The resulting spiking is useable data, although the autoscaling time-dependency reduces the utility of the absolute value. In general, the patterns observed in the satellite and glider data sets were in agreement; however, the inter-glider calibration problems, long-term drift, and fouling suggests caution is required in use of the fluorescence data.

The crude oil CDOM and turbidity sensors both were compromised to some degree by overriding signals. Solar induced fluorescence prevented use of diurnal data in the CDOM sensor, and bubbles appeared to be a significant (and unquantifiable) problem for the turbidity sensor. Placement of the turbidity sensor in the sub body 7 m below the surface would likely have minimized this problem.

Platform fouling is a nearly unavoidable consequence of the long-term deployment of these 4 Wave Gliders. The optical sensors appeared to be more subject to their presumptive effects than did the sensors for temperature and salinity. The inability to determine whether a signal was real or an artifact limited the use of some of the data and suggests that redundant systems are required for very long term deployments. In addition, a capability to image the sensor heads on command would significantly reduce the uncertainty as to the cause of drift and noise.

While the systems do not capture three-dimensional data as Argos floats or profiling autonomous vehicles such as the Slocum glider can, the immense duration of the missions and high resolution surface mapping potential of the Wave Glider is clearly evident in the detail captured in the equatorial crossing. The systems are directly analogous to surface vessels’ underway sampling systems and as size, cost and power requirements of sensors continue to drop, additional uses will develop. In particular, the systems appear useful for calibrating satellite data and targeting the temperature-salinity surface characteristics of oceanographic fronts and water masses. While these 4 glider missions were not designed to test specific hypotheses and or to calibrate satellite data, the data sets have demonstrated the Wave Gliders ability to sample known features along great distances. Within the data sets are some indicators of unusual properties that merit further examination.

The continuous monitoring of biological properties captured the characteristics of phytoplankton in different oceanographic provinces. The diel fluorescence pattern of phytoplankton has been well documented [48]–[50], [53], [54] and both the *Papa Mau* and *Benjamin* captured the transition from the gyre to the equatorial current system. The timing of the pre-dawn fluorescence decrease was notably different in the two water masses. Behernfeld et al [55] noted a transition from N limitation to Fe limitation at ∼5° N linked to the equatorial/subtropical transition by using advanced active fluorescence systems to monitor the characteristics of normalized variable fluorescence. This is much more sophisticated than the Turner C3 measurements, but if power requirements can be suitably reduced, the Wave Gliders would be able to provide a novel mapping ability for the ocean that could advance our understanding of both basin-scale and regional patterns. In particularly, the impact of sub-mesoscale interactions on phytoplankton growth and biomass development [47], [56], [57] would become accessible at daily the time- scale relevant to these processes.

The event sampled by the *Piccard Maru* in August 2012 is consistent with phytoplankton aggregations. Several features are noteworthy. The event occurred at the interaction of two mesoscale features, a cyclonic and anticylonic feature similar to that noted well to the east[47]. In these regions, zones of horizontal turbulent stirring are created that lead to localized ageostrophic flows and the resultant production of upwelling and downwelling zones [56], [57]. In addition, these eddies inject, on average, 88 mmol N m^−2^ y^−1^ into the Pacific gyre near Hawaii [58] due to nutricline uplift and may enhance localized blooms of nitrogen-fixing phytoplankton [59] via the submesoscale vertical transport of nutrients. Guidi et al [47] directly observed the impact on *Trichodesmium* communities, and documented the accumulation and enhanced export of *Trichodesmium* colonies in the frontal zone between the eddies and at the edge of the anticyclone. In their study, they sampled across an elevated chlorophyll feature at the interface of a cyclone and anticyclone, analogous to the features noted north and east of the glider track in Fig. 11c and directly sampled on 1 Aug 2011 at ∼180 °E. A slight elevation in the fluorometer fluorescence during the major aggregation event suggests this was real. The aggregation event also coincided with a salinity increase and location consistent with the southern subtropical front [36]. Diatom symbiosis commonly bloom along the northern subtropical front [24], and the lack of a satellite signal is more consistent with a *Hemiaulus* bloom [41] than *Trichodesmium*. The data is not conclusive although it does highlight the role Wave Gliders could play in tracking these blooms if properly instrumented.

Measurements in this top layer of the ocean are difficult and not commonly made. Fresh surface salinity lens associated with rainfall have been observed with thicknesses of several meters [45], [60] and they can extend to as deep as 40 m [31], [61], [62] These “rain puddles” are generally characterized as being small scale, with horizontal scales of ∼10 km [45]. This small-scale heterogeneity is evident in the glider salinity data as the numerous spikes of lower salinity, particularly during periods of rainfall. However an interesting result of the Pac-X mission is the observation that shallow lenses of fresh water may occur over much larger spatial scales.

## Conclusions

The Wave Glider PacX missions documented the long-deployment capabilities of the systems for examining oceanographic patterns and distributions. The temperature and salinity sensors were stable; the optical sensors generated data that requires careful evaluation prior to use. Surface based measurements are highly sensitive to solar radiation and bubbles; sensor selection, design and location on the gliders are critical to successful data recovery. Autogain features, while necessary, complicated specific analyses of the fluorescence data for aggregation phenomena. Redundant sensors and sensor head imaging would be useful to support the data analysis and help interpret patterns in cases of long-term drift. Fouling is a problem in multiple month deployments although manageable. The systems have capability to provide surface validation of satellite measurements, and to track fronts and rapidly developing features particularly if multiple gliders can be used. The ability to track diel changes in phytoplankton physiology suggests an expanded role for them in mapping nutrient limitation in these communities. They can survive tropical cyclone conditions.

Notable features observed in these missions are indications of western Pacific low salinity lenses and an apparent phytoplankton aggregation event. Differences in the Aquarius sea surface salinity data and glider-based salinity sensor revealed the existence of low salinity surface pools in the western Pacific. Examination of the chl fluorescence data suggested a substantial aggregation event occurred in the frontal zone between two mesoscale eddies in the western Pacific. It is consistent with either a *Trichodesmium* accumulation or diatom aggregates. The sensitivity to diel rhythms in chlorophyll fluorescence suggests physiological information is possible to obtain with the appropriate sensors.

## Supporting Information

Figure S1 **Photodocumentation of** ***Benjamin’s*** **C3 sensor head upon recovery in Australia.**
(PDF)

Figure S2 **Photodocumention of** ***Piccard Maru*** **bottom fouling at the Hawaii recovery.**
(PDF)

Figure S3 **Photodocumentation of** ***Fontaine Maru*** **.** A. Hawaii recovery. The circular C3 sensor head can be seen at the left of the glider. B. After recovery in the western Pacific.
(PDF)

Figure S4 **Complete mission data plot for** ***Piccard Maru.***
(PDF)

Figure S5 **Complete mission data plot for the** ***Fontaine Maru*** **.**
(PDF)

Figure S6 **Hurricane tracking plot and glider** ***Benjamin*** **track**. Purple symbols indicate position at 1200 UTC on 31 Dec. 2012.
(TIF)

Figure S7 **Diel pattern of chl fluorescence and oil CDOM from the equatorial Pacific**. A. chl fluorescence, B. oil CDOM. Position of glider *Benjamin* on 12 August 2012: 1.511 °S 170.621 °W. Dark bars indicate night time.
(TIF)

Figure S8 **Salinity plots in the eastern N. Pacific gyre**. Data is pooled from all 4 gliders. Letters A, B indicate the crossing of the subtropical front, C indicates a salinity front at the southern boundary of the high salinity gyre water.
(TIF)

Text S1 (PDF)
