# Correction: A Comparison of the Pac-X Trans-Pacific Wave Glider Data and Satellite Data (MODIS, Aquarius, TRMM and VIIRS)

**DOI:** 10.1371/journal.pone.0096968

**Published:** 2014-04-29

**Authors:** The PLOS ONE Staff

The authors would like to provide a higher quality image for Figure 7. Please view the new Figure 7 here.

**Figure 7:**
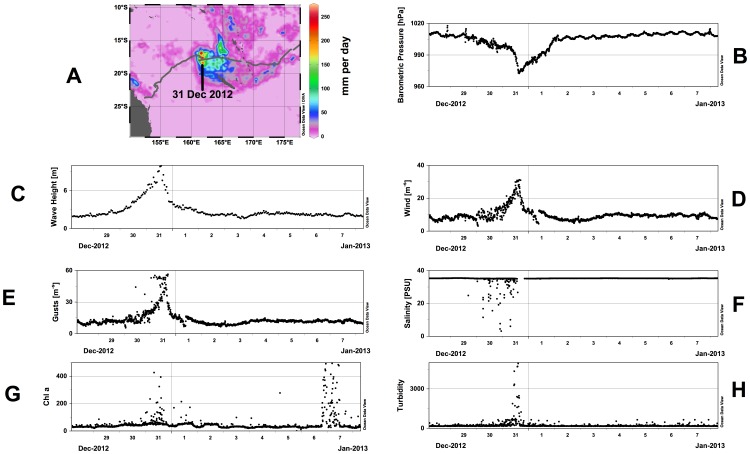
Tropical Storm Freda. (a) Glider track overlaid on TRMM precipitation estimate for 31 Dec. 2012. (b) Barometric pressure. (c) Significant wave height. (d) Average wind speed. (e) Maximum wind speed. (f) Salinity. (g) Chlorophyll a fluorescence. (h) Turbidity.
