# Supplementary figures and images for: A Comparison of the Pac-X Trans-Pacific Wave Glider Data and Satellite Data (MODIS, Aquarius, TRMM and VIIRS)
Source: PLoS One. 2014 Mar 21;9(3):e92280. doi: 10.1371/journal.pone.0092280 (PMC3962394; doi:10.1371/journal.pone.0092280)

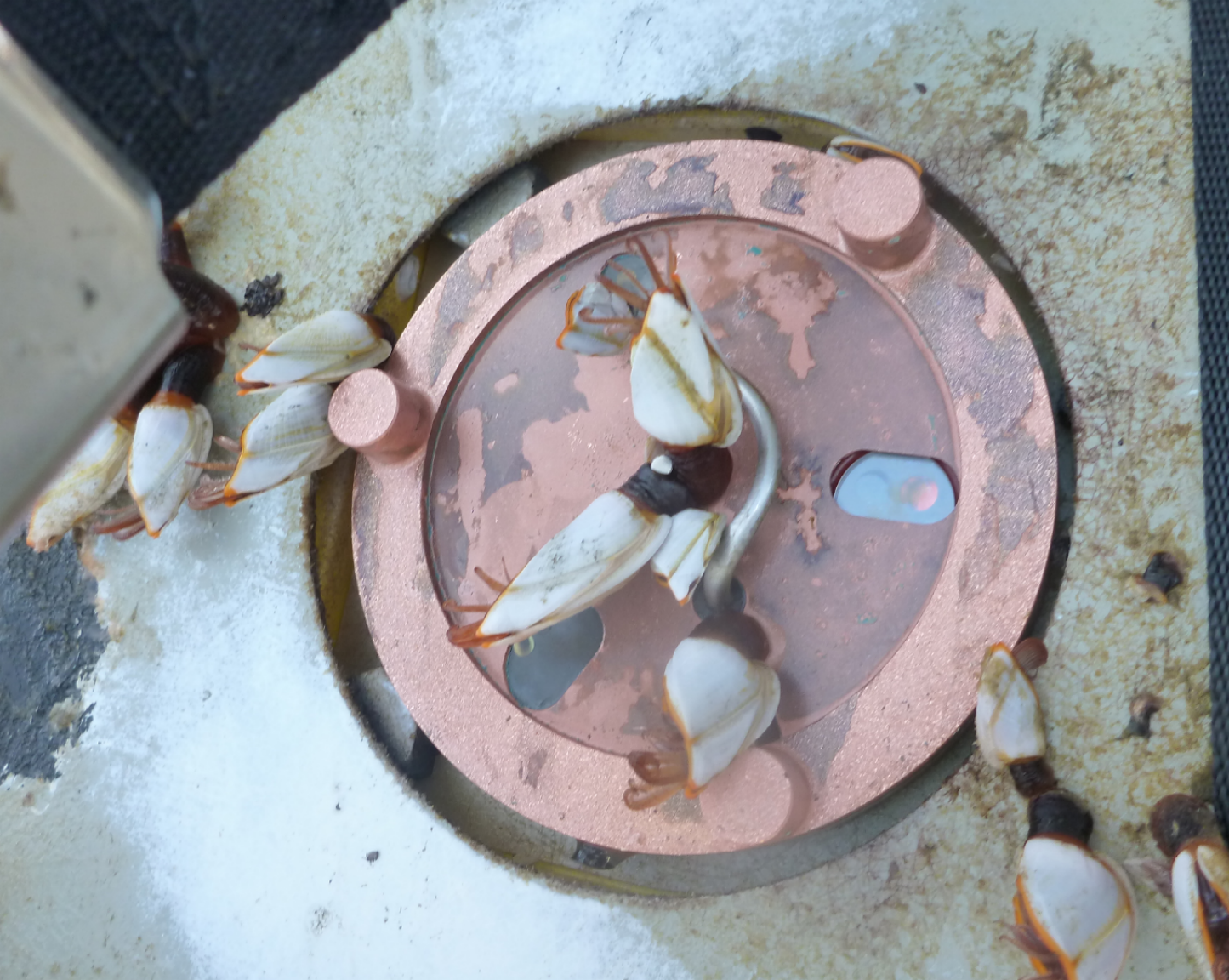

Supplement: Figure S1 — Photodocumentation of Benjamin’s C3 sensor head upon recovery in Australia. (PDF) [file pone.0092280.s001.pdf]

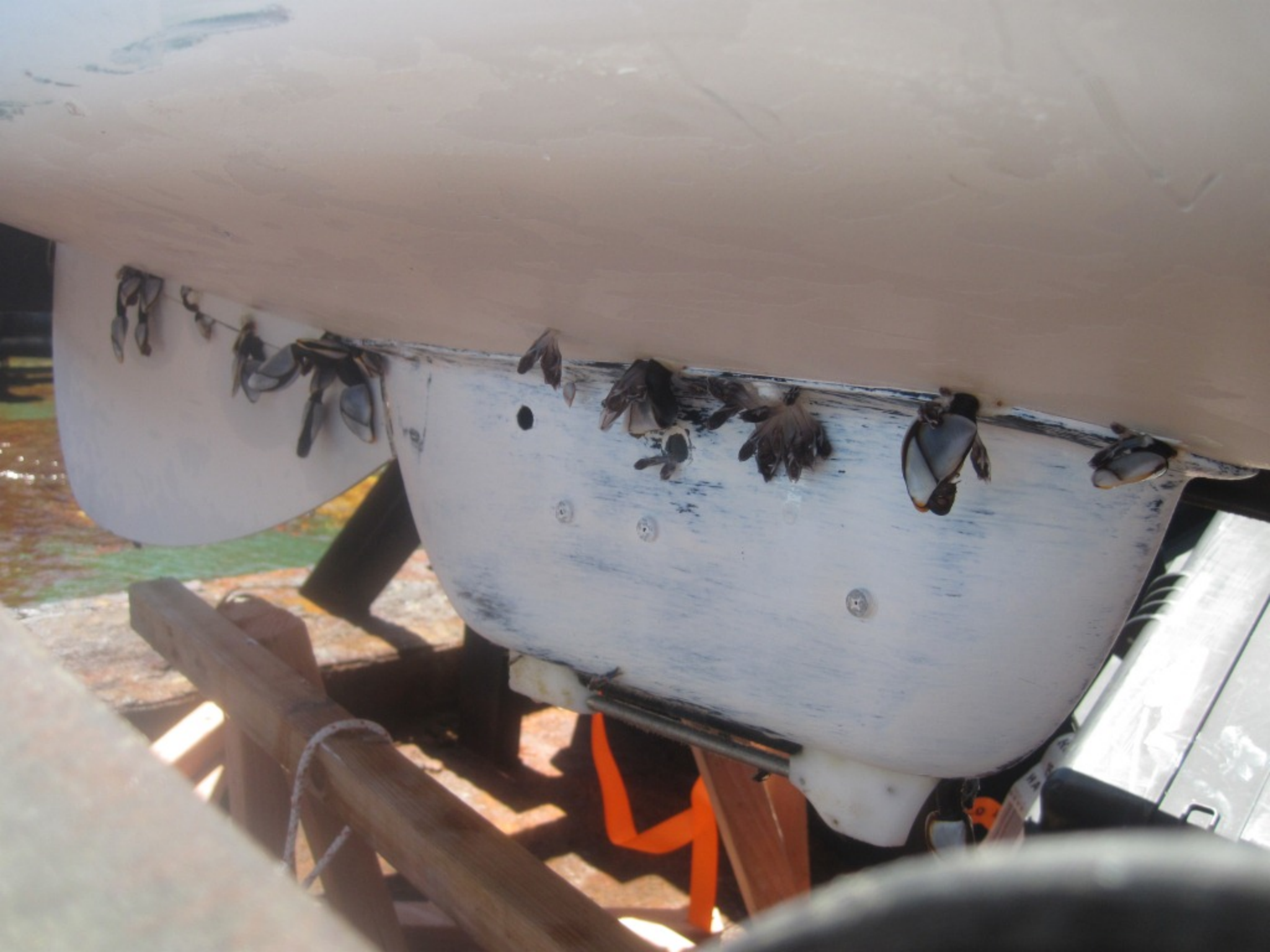

Supplement: Figure S2 — Photodocumention of Piccard Maru bottom fouling at the Hawaii recovery. (PDF) [file pone.0092280.s002.pdf]

# Piccard Maru

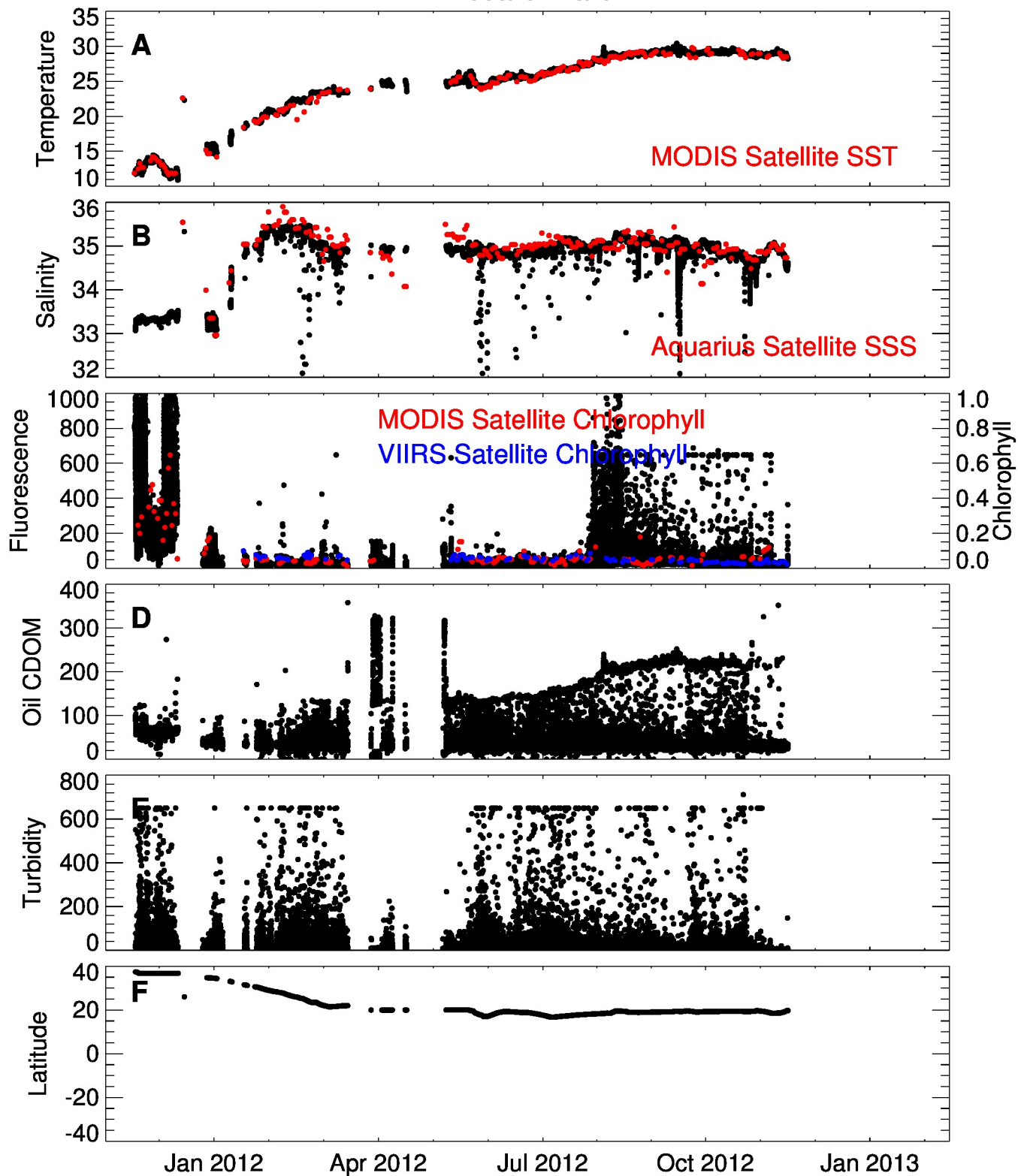

Supplement: Figure S4 — Complete mission data plot for Piccard Maru. (PDF) [file pone.0092280.s004.pdf]

# Fontaine Maru

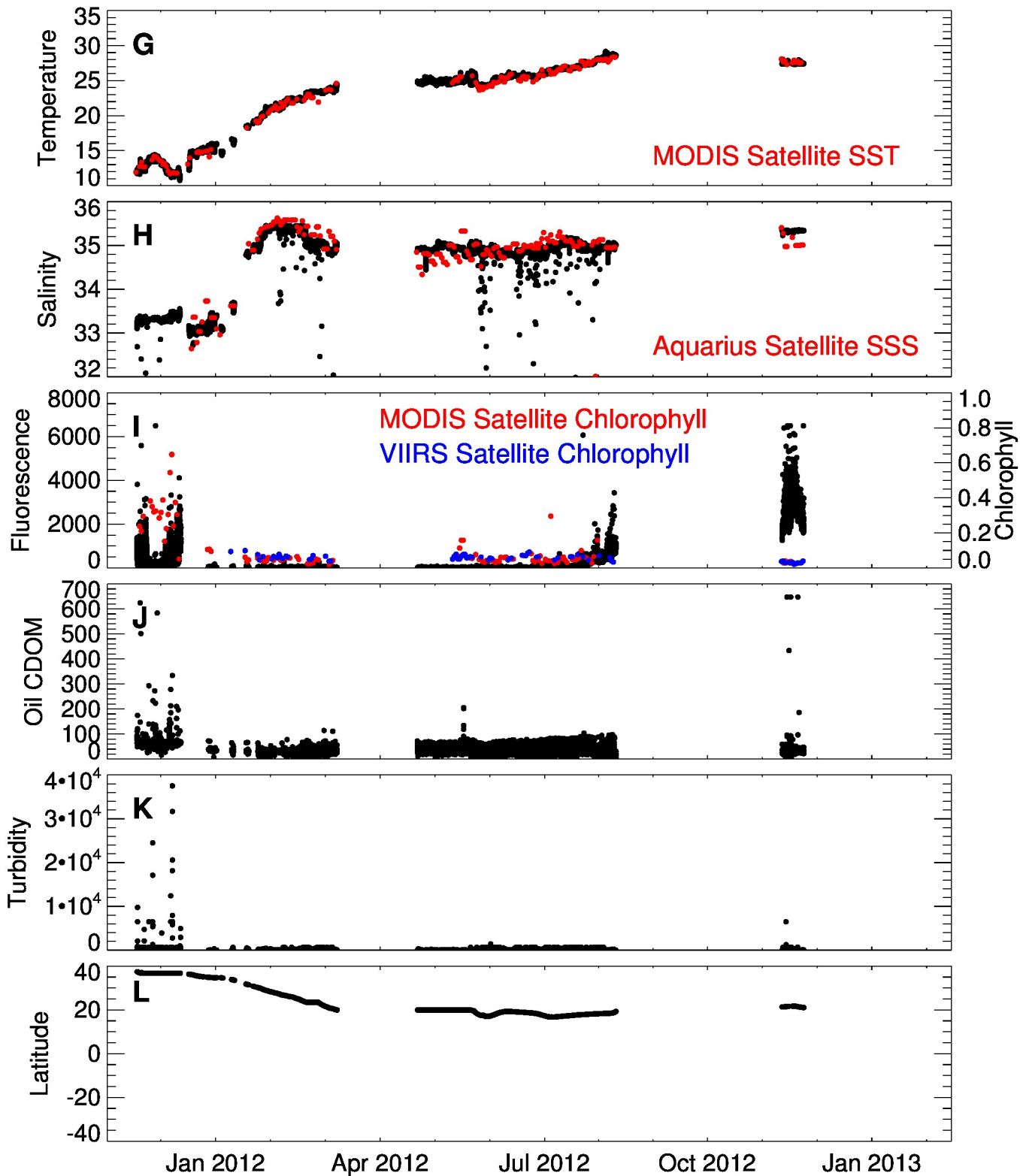

Supplement: Figure S5 — Complete mission data plot for the Fontaine Maru . (PDF) [file pone.0092280.s005.pdf]

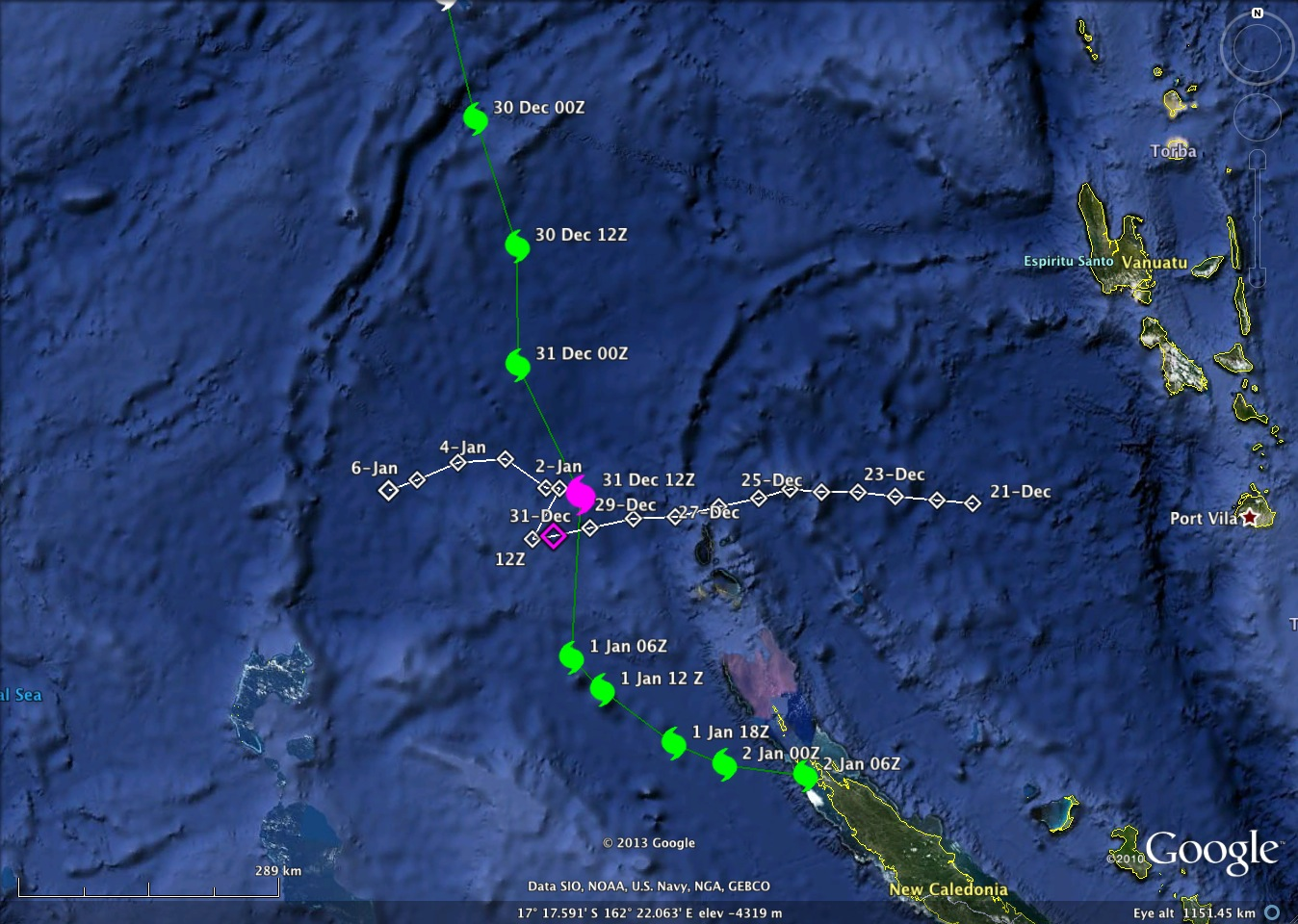

Supplement: Figure S6 — Hurricane tracking plot and glider Benjamin track. Purple symbols indicate position at 1200 UTC on 31 Dec. 2012. (TIF) [file pone.0092280.s006.tif]

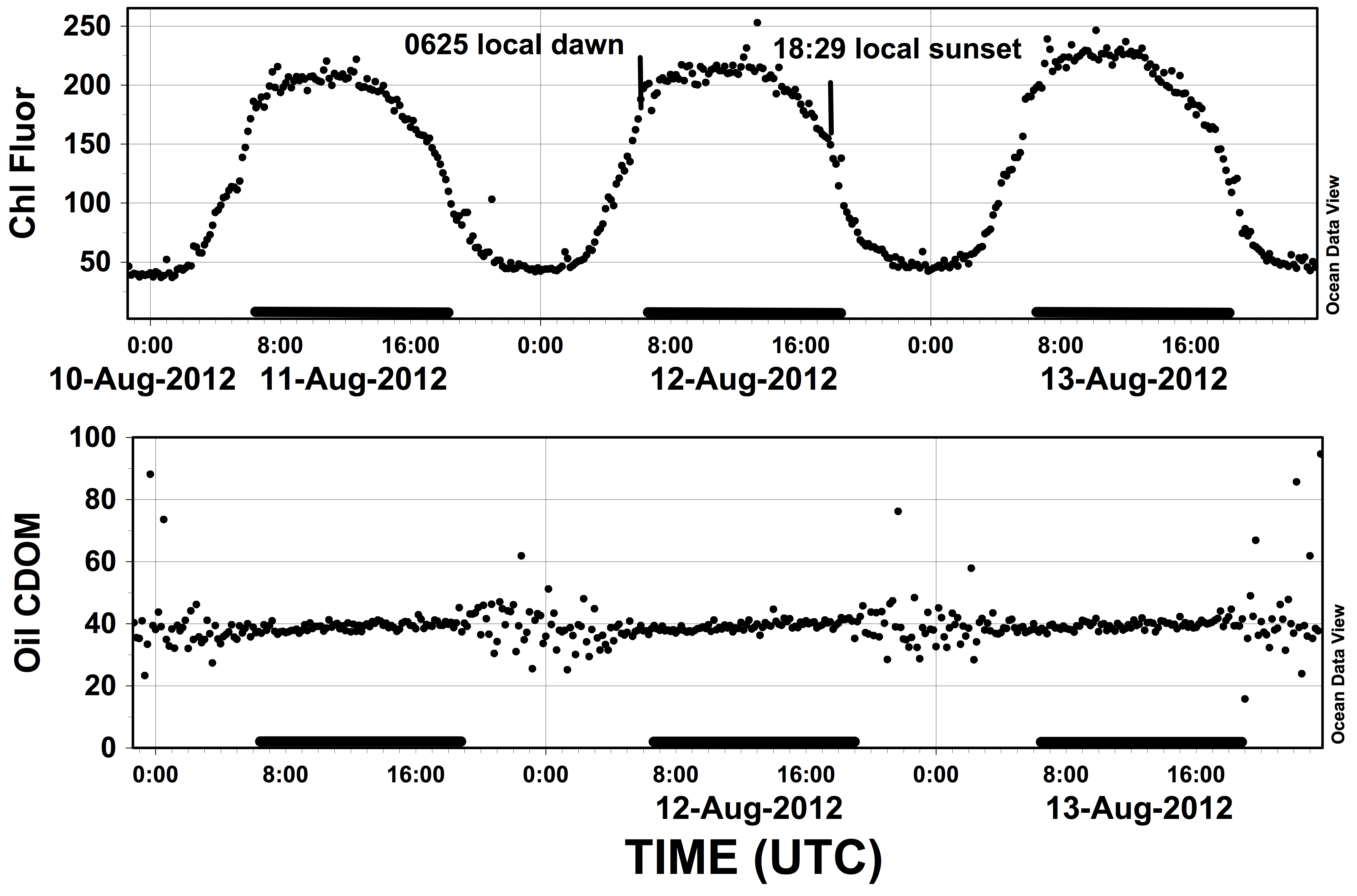

Supplement: Figure S7 — Diel pattern of chl fluorescence and oil CDOM from the equatorial Pacific. A. chl fluorescence, B. oil CDOM. Position of glider Benjamin on 12 August 2012: 1.511 °S 170.621 °W. Dark bars indicate night time. (TIF) [file pone.0092280.s007.tif]

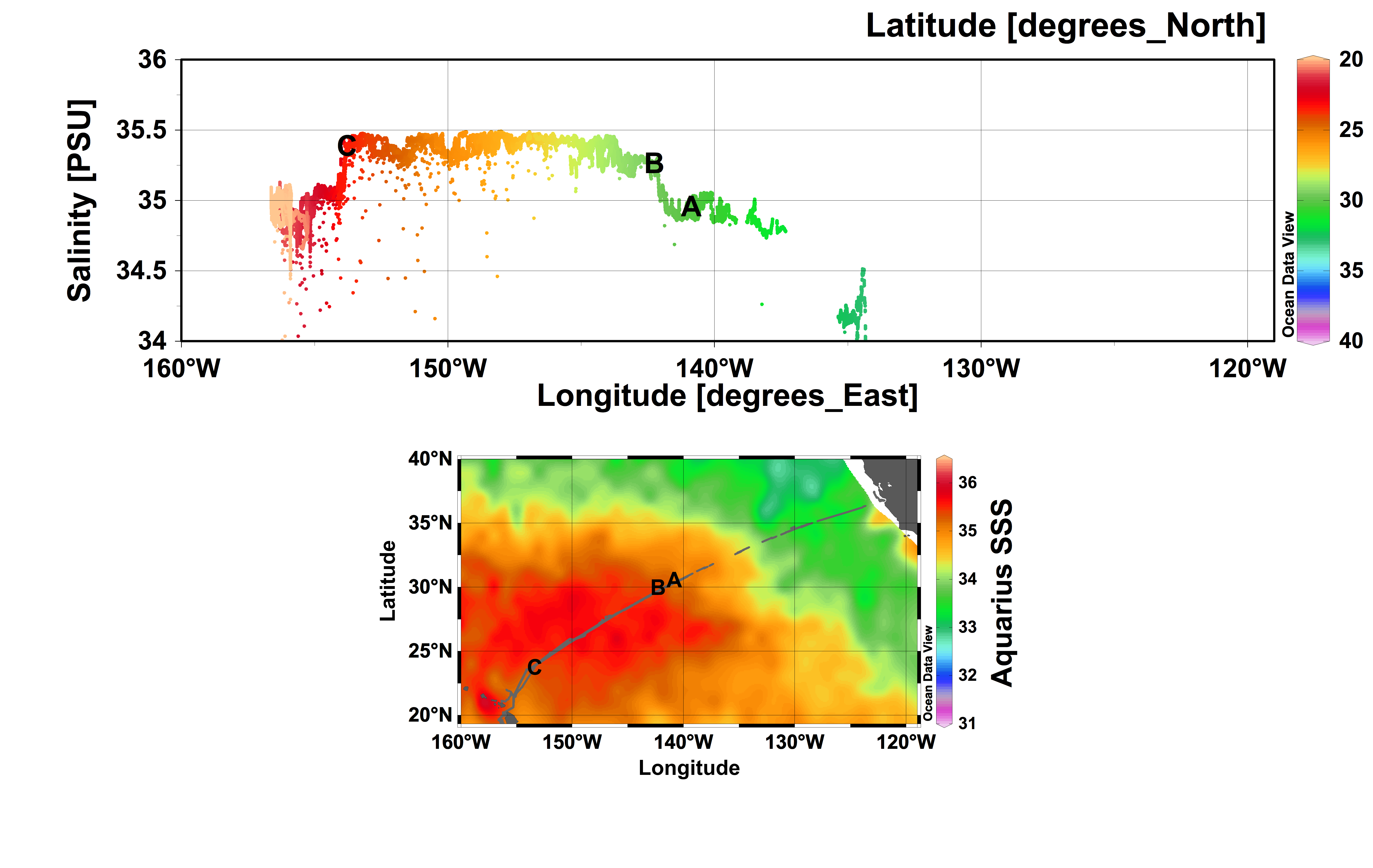

Supplement: Figure S8 — Salinity plots in the eastern N. Pacific gyre. Data is pooled from all 4 gliders. Letters A, B indicate the crossing of the subtropical front, C indicates a salinity front at the southern boundary of the high salinity gyre water. (TIF) [file pone.0092280.s008.tif]
