# Supplementary material for: A Comparison of the Pac-X Trans-Pacific Wave Glider Data and Satellite Data (MODIS, Aquarius, TRMM and VIIRS)
Source: PLoS One. 2014 Mar 21;9(3):e92280. doi: 10.1371/journal.pone.0092280 (PMC3962394; doi:10.1371/journal.pone.0092280)

Figure S3. Photodocumentation of *Fontaine Maru*. A. Hawaii recovery. The circular C3 sensor head can be seen at the left of the glider. B. After recovery in the western Pacific..

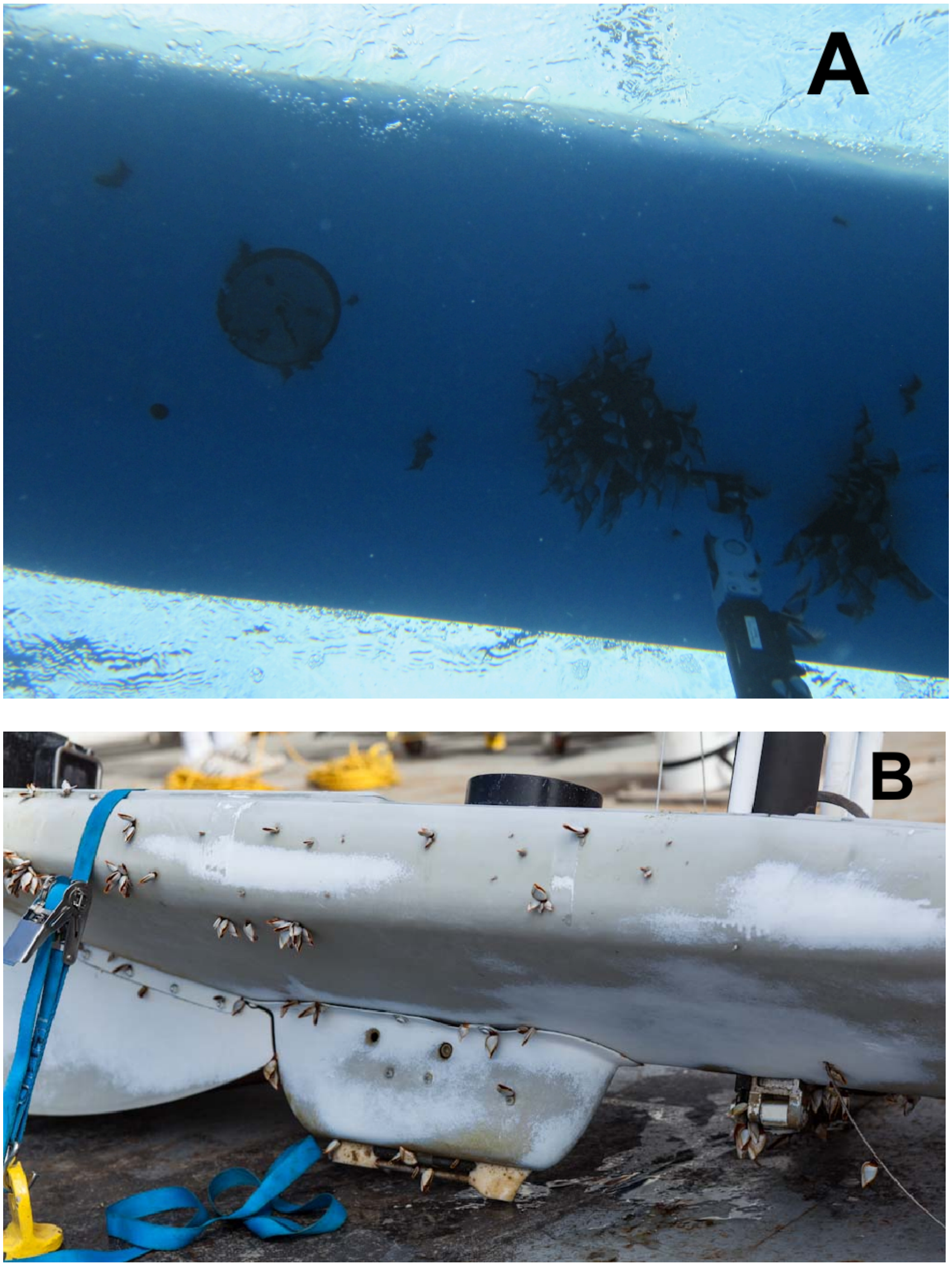

Supplement: Figure S3 — Photodocumentation of Fontaine Maru . A. Hawaii recovery. The circular C3 sensor head can be seen at the left of the glider. B. After recovery in the western Pacific. (PDF) [file pone.0092280.s003.pdf]
