# Supplementary material for: A Comparison of the Pac-X Trans-Pacific Wave Glider Data and Satellite Data (MODIS, Aquarius, TRMM and VIIRS)
Source: PLoS One. 2014 Mar 21;9(3):e92280. doi: 10.1371/journal.pone.0092280 (PMC3962394; doi:10.1371/journal.pone.0092280)
Supplement: Text S1 — (PDF) [file pone.0092280.s009.pdf]

Supplemental Text

Wave Glider calibration data files for the Turner C3 fluorometers as provided by Liquid Robotics. The authors played no role in pre- or post- mission calibration

PacX Fluorometer Calibration

Pac X Vehicle	Papa Mau	Benjamin	Piccard Maru	Fontaine Maru
C3 Serial #	0315	0241	0230	0316

All four of these fluorometers were calibrated in the LRI sensor lab on September 28th, 2011. All instruments had identical channels.

Fontaine Maru (0316) was recovered and the calibration rechecked on February 13, 2013.

Papa Mau (0315) was recovered and the calibration rechecked on February 18, 2013.

Benjamin (0241) was recovered and the calibration rechecked on March 22, 2013

Channel	Reagent
1) Crude Oil	Quinine Sulfate(250 mg/L)
2) chl-A	Basic Blue 3(1.0119 mg/mL)
3) Turbidity	Turbidity Standard(3000 NTU)

PacX Fluorometer Calibration
Crude Oil Channel

Serial Number	Date	Y-intercept	Slope rfu/ppb	Date	Y-intercept	Slope rfu/ppb
0315	09/28/2011	85.79	0.55	02/18/2013	33.91	0.81[(1)]
0241	09/28/2011	86.44	0.61	03/22/2013	44.32	1.06[(1)]
0230[(2)]	09/28/2011	92.54	0.62			
0316	09/28/2011	88.08	0.62	2/13/2013	41.93	0.54

(1): See Notes
(2): See Notes

PacX Fluorometer Calibration Chlorophyll-A Channel

Serial Number	Date	Y-intercept	Slope rfu/ppb	Date	Y-intercept	Slope rfu/ppb
0315	09/28/2011	18.8	7.73	02/18/2013	106.2	31.4(3)
0241	09/28/2011	22.7	8.79	03/22/2013	188.4	37.6(3)
0230	09/28/2011	2.4	8.50			
0316	09/28/2011	44	6.62	02/13/2013	89.5	22.5(3)

(3): See Notes

PacX Fluorometer Calibration
Turbidity Channel

Serial Number	Date	Y-intercept	Slope rfu/ntu	Date	Y-intercept	Slope rfu/ntu
0315	09/28/2011	13.39	20.73	02/18/2013	5.60	21.00
0241	09/28/2011	17.65	20.02	03/22/2013	13.2	19.52
0230	09/28/2011	15.36	22.26			
0316	09/28/2011	13.15	20.67	02/13/2013	3.27	0.15[(4)]

(4): See Notes

NOTES

(1) **Oil**

The post-deployment slopes for serial numbers 0241 and 0315 were higher when compared to the pre-deployment slopes. Because of the typical degradation of optical instruments in the field, this result is unlikely. For ease of use, the quinine sulfate solution was made up using water in place of the recommended sulfuric acid. The stability of QS is unknown when prepared in this manner and is a likely source of the discrepancy. As an alternative to our calibration, both instruments were sent to Turner for testing to check for any degradation in response. Below are the results of the testing performed at Turner.

Serial Number	Date	Gain	Date	Gain	Percent Change
0241	09/2011	274.32mv	04/2013	297.45mv	+8
0315	09/2011	255.95mv	04/2013	270.17mv	+6

Turner Designs considers these units to be unchanged. The target gain for Turner is ~270mv with a range of 240-300mv

NOTES

(2) Serial number 0230 has not been recovered.

(3) **Chlorophyll-a**

The Basic Blue powder used to make up the post deployment calibration standard is 25% dye content. The concentration of the pre-deployment calibration standard was labeled as 1.0119 mg/ml. The post-deployment calibration standard was mixed to match the concentration of the pre-deployment standard but when comparing the post deployment and pre-deployment slopes, the post deployment values were roughly 4 times. This condition was observed across all 3 chl-A channels. The most likely explanation for this discrepancy is that the original solution could have been a concentration of 0.253 mg/ml (0.25 * 1.0119 mg/ml) on account of the technician not being aware of the 25% dye content.

NOTES

Chlorophyll-A Channel

Slope values in red are assuming that the basic blue concentration was 0.253mg/ml for the pre-deployment calibration

Serial Number	Date	Y-intercept	Slope rfu/ppb	Date	Y-intercept	Slope rfu/ppb
0315	09/28/2011	18.8	30.9	2/18/2013	106.2	31.4
0316	09/28/2011	44	26.5	2/13/2013	89.5	22.5
0241	09/28/2011	22.7	35.2	3/22/2013	188.4	37.6

NOTES

(4) **Turbidity**

The turbidity channel for C3 serial number 0316 had little to no response during the post-deployment calibration. While we can't be absolutely certain, this is likely due to the removal of the instrument from the vehicle. The anti-fouling copper plates fused together and we had to cut them off the instrument. The data were reviewed for evidence of a catastrophic failure but nothing was found.

Calibration drift reports are included as reported by Seabird Electronics. Sensor serial numbers are as follows. The *Piccard Maru* was not recovered. Detailed calibration files are available in the NODC archive listed in the Methods and Materials.

Seabird Sensor	Glider
0040	*Fontaine Maru*
0041	*Benjamin*
0042	*Papa Mau*
0039	*Piccard Maru*

Table S1. Sensor specifications for the Turner C3 and Seabird gpCTD. Information is from the manufacturer's website.

Sensor	Detection Limit	Accuracy	Range	Resolution
Chl in vivo	0.025 µg/L	-	0-500 µg/L	-
Crude Oil	0.2 ppb -	0-2700 ppb	-	
Turbidity	0.05 NTU	-	0-3000 NTU	-
Conductivity	-	±0.003 mS/cm	0-60 mS/cm	0.0001 mS/cm
Temperature	-	±0.004 °C	+1-32°C	0.001 °C

SBE SEA-BIRD ELECTRONICS, INC.
13431 NE 20th St. Bellevue, Washington 98005 USA
Phone: (425) 643-9866 Fax: (425) 643-9954 www.seabird.com

Temperature Calibration Report

Customer:	Liquid Robotics		
Job Number:	72981	Date of Report:	3/29/2013
Model Number	Glider	Serial Number:	0040 Payload Glider

Temperature sensors are normally calibrated 'as received', without adjustments, allowing a determination sensor drift. If the calibration identifies a problem, then a second calibration is performed after work is completed. The 'as received' calibration is not performed if the sensor is damaged or non-functional, or by customer request.

An 'as received' calibration certificate is provided, listing coefficients to convert sensor frequency to temperature. Users must choose whether the 'as received' calibration or the previous calibration better represents the sensor condition during deployment. In SEASOFT enter the chosen coefficients. The coefficient 'offset' allows a small correction for drift between calibrations (consult the SEASOFT manual). Calibration coefficients obtained after a repair apply only to subsequent data.

'AS RECEIVED CALIBRATION' ☑ Performed ☐ Not Performed

Date: 3/9/2013 Drift since last cal: -0.00009 Degrees Celsius/year

Comments:

'FINAL CALIBRATION' ☑ Performed ☐ Not Performed

Date: 3/29/2013 Drift since 07 Sep 11 +0.00042 Degrees Celsius/year

Comments:

SBE
SEA-BIRD ELECTRONICS, INC.
13431 NE 20th Street Bellevue, Washington 98005 USA
Phone: (425) 643-9866 Fax: (425) 643-9954 www.seabird.com

Conductivity Calibration Report

**Customer:**	Liquid Robotics		
**Job Number:**	72981	**Date of Report:**	3/29/2013
**Model Number**	Glider	**Serial Number:**	0040 Payload Glider

Conductivity sensors are normally calibrated 'as received', without cleaning or adjustments, allowing a determination of sensor drift. If the calibration identifies a problem or indicates cell cleaning is necessary, then a second calibration is performed after work is completed. The 'as received' calibration is not performed if the sensor is damaged or non-functional, or by customer request.

An 'as received' calibration certificate is provided, listing the coefficients used to convert sensor frequency to conductivity. Users must choose whether the 'as received' calibration or the previous calibration better represents the sensor condition during deployment. In SEASOFT enter the chosen coefficients. The coefficient 'slope' allows small corrections for drift between calibrations (consult the SEASOFT manual). Calibration coefficients obtained after a repair or cleaning apply only to subsequent data.

'AS RECEIVED CALIBRATION' ☑ Performed ☐ Not Performed

Date: 3/9/2013 Drift since last cal: +0.00210 PSU/month

Comments:

'CALIBRATION AFTER REPAIR' ☑ Performed ☐ Not Performed

Date: 3/29/2013 Drift since Last cal: N/A PSU/month

Comments:
The conductivity cell was replaced.

**Measured at 3.0 S/m*

Cell cleaning and electrode replatinizing tend to 'reset' the conductivity sensor to its original condition. Lack of drift in post-cleaning-calibration indicates geometric stability of the cell and electrical stability of the sensor circuit.

SBE
SEA-BIRD ELECTRONICS, INC.
13431 NE 20th St. Bellevue, Washington 98005 USA
Phone: (425) 643-9866 Fax: (425) 643-9954 www.seabird.com

Temperature Calibration Report

**Customer:**	Liquid Robotics		
**Job Number:**	73338	**Date of Report:**	4/4/2013
**Model Number**	Glider	**Serial Number:**	0042 Payload Glider

Temperature sensors are normally calibrated 'as received', without adjustments, allowing a determination sensor drift. If the calibration identifies a problem, then a second calibration is performed after work is completed. The 'as received' calibration is not performed if the sensor is damaged or non-functional, or by customer request.

An 'as received' calibration certificate is provided, listing coefficients to convert sensor frequency to temperature. Users must choose whether the 'as received' calibration or the previous calibration better represents the sensor condition during deployment. In SEASOFT enter the chosen coefficients. The coefficient 'offset' allows a small correction for drift between calibrations (consult the SEASOFT manual). Calibration coefficients obtained after a repair apply only to subsequent data.

'AS RECEIVED CALIBRATION' ☑ Performed ☐ Not Performed

Date: 3/30/2013 Drift since last cal: -0.00004 **Degrees Celsius/year**

Comments:

'FINAL CALIBRATION' ☑ Performed ☐ Not Performed

Date: 4/4/2013 Drift since 19 Sep 11 +0.00001 **Degrees Celsius/year**

Comments:

SEA-BIRD ELECTRONICS, INC.
13431 NE 20th Street Bellevue, Washington 98005 USA
Phone: (425) 643-9866 Fax: (425) 643-9954 www.seabird.com

Conductivity Calibration Report

**Customer:**	Liquid Robotics		
**Job Number:**	73338	**Date of Report:**	4/4/2013
**Model Number**	Glider	**Serial Number:**	0042 Payload Glider

Conductivity sensors are normally calibrated 'as received', without cleaning or adjustments, allowing a determination of sensor drift. If the calibration identifies a problem or indicates cell cleaning is necessary, then a second calibration is performed after work is completed. The 'as received' calibration is not performed if the sensor is damaged or non-functional, or by customer request.

An 'as received' calibration certificate is provided, listing the coefficients used to convert sensor frequency to conductivity. Users must choose whether the 'as received' calibration or the previous calibration better represents the sensor condition during deployment. In SEASOFT enter the chosen coefficients. The coefficient 'slope' allows small corrections for drift between calibrations (consult the SEASOFT manual). Calibration coefficients obtained after a repair or cleaning apply only to subsequent data.

'AS RECEIVED CALIBRATION' ☑ Performed ☐ Not Performed

Date: 3/30/2013 Drift since last cal: +0.00200 PSU/month*

Comments:

'CALIBRATION AFTER REPAIR' ☑ Performed ☐ Not Performed

Date: 4/4/2013 Drift since Last cal: N/A PSU/month*

Comments:
The conductivity cell was replaced.

****Measured at 3.0 S/m***

Cell cleaning and electrode replatinizing tend to 'reset' the conductivity sensor to its original condition. Lack of drift in post-cleaning-calibration indicates geometric stability of the cell and electrical stability of the sensor circuit.

Supplemental Text.

This is the text of an email from Luke Beatman, Liquid Robotics concerning data acquisition in Exclusive Economic Zones

Luke Beatman <luke.beatman@liquidr.com>

To: Tracy Villareal <tracyv@austin.utexas.edu>

Received: from ironwood.mail.utexas.edu (146.6.25.5) by EXHUB01.austin.utexas.edu (172.16.71.86) with Microsoft SMTP Server (TLS) id 14.2.342.3; Wed, 1 May 2013 07:38:35 -0500

Received: from na3sys009aog128.obsmtp.com ([74.125.149.141]) by ironwood.mail.utexas.edu with SMTP; 01 May 2013 07:38:32 -0500

Received: from mail-we0-f199.google.com ([74.125.82.199]) (using TLSv1) by na3sys009aob128.postini.com ([74.125.148.12]) with SMTP ID DSNKUYEMx6nVG+6EcW53XEqw+bQxRbQFI/d9@postini.com; Wed, 01 May 2013 05:38:32 PDT

Received: by mail-we0-f199.google.com with SMTP id x43so1933035wey.6 for <tracyv@austin.utexas.edu>; Wed, 01 May 2013 05:38:30 -0700 (PDT)

Received: by 10.223.4.218 with HTTP; Wed, 1 May 2013 05:38:29 -0700 (PDT)

X-Utexas-Sender-Group: None

X-Ironport-Mid: 532302894

X-Sbrs: 4.8

X-Ironport-Anti-Spam-Filtered: true

X-Ironport-Anti-Spam-Result:
AuICAMoLgVFKfZWNlGdsb2JhbABPAw6CNcARdR4OAQEBAQcNCQkUBSOCHwEBAQMB
DwFuCwsEBzsiEgEFARwGE4gGBqJAinABAZFjjwkXEYM/A4kXjg+PSBYpg3Rf

X-Ironport-Av: E=Sophos;i="4.87,588,1363150800"; d="scan'208";a="532302894"

X-Utexas-Seen-Inbound: true

X-Google-Dkim-Signature: v=1; a=rsa-sha256; c=relaxed/relaxed; d=google.com; s=20120113; h=x-received:mime-version:x-received:in-reply-to:references:date :message-id:subject:from:to:content-type:x-gm-message-state;
bh=kONAV845TjcrCFcBMhgLfCcM/mgJshFIV0lZahbOaJM=;
b=nhZbdc0KQQSwSUNO4V2rbdsrTwLA6+My/eWVT5Ll5UBeHOEwyJ1sA6nagIkSuROZgq
gNLj4aFUeyNA5TWeSld79CzYNMR+OhIZ2tG+fUX0AE30xQDP/mC9l3wW0MtiRie7x21H
lTcDlh1JFGU45U8CAAsqcgwxr0aBht+51l/0Hm0IfLzDNHWRqiSjfuTneR+8EI7SSuS2
i6CpVEMzkBzCSMJTC/Ky9Jow6ByZ4LqXUKkGU0jF8vvcd3+tsUswTzogTprSuNs9+cHj
AAe3sGGekxc1gaNIGWbYgNY/V1zVbwPinytEppEZWkqGmuGQyt9f1Ly6coV5ef7rz30+
WdcQ==

X-Received: by 10.15.98.66 with SMTP id bi42mr7495283eeb.39.1367411910208; Wed, 01 May 2013 05:38:30 -0700 (PDT)

X-Received: by 10.15.98.66 with SMTP id bi42mr7495266eeb.39.1367411910114; Wed, 01 May 2013 05:38:30 -0700 (PDT)
In-Reply-To: <0F21B2DB-1888-4B5B-9BC3-C8DFBD3EF3F8@austin.utexas.edu>
References: <02C607A3-A895-42C7-84CC-AD6CAECA302B@austin.utexas.edu>
<CADN0qyALapZYde_YxjF73g9DcLgLV9_qh3C6t2VQvoY2nqo+kw@mail.gmail.com>
<0F21B2DB-1888-4B5B-9BC3-C8DFBD3EF3F8@austin.utexas.edu>
Message-Id: <CADN0qyDFXov5fMVO2NxUWkz8MVTd=gxNsXSdE=HK0MDH-2sHrA@mail.gmail.com>
Content-Type: multipart/alternative; boundary="089e016815b62a23f304dba76516"
X-Gm-Message-State:
ALoCoQlLjfZmMwQ+/+Tb+/ckAfAm2jyMYrz2KxZzGBtXGEIc7ZIe8NKe9WGEeThqNh4XwM+fYIj/3uDCIsDXCa+Ew3NbHuZ0JvPJ4KfeN5/9TSrVWIpGq8Voc5Mc+Bp1sPPg3RI2Spz09LUOwogxpM0nreHMQJgRgc/UEuVJDG2ObDq9cE3V30c=
Return-Path: luke.beatman@liquidr.com
X-Ms-Exchange-Organization-Authsource: EXHUB01.austin.utexas.edu
X-Ms-Exchange-Organization-Authas: Anonymous
X-Ms-Exchange-Organization-Avstamp-Mailbox: MSFTFF;1;0;0 0 0
Mime-Version: 1.0
Re: question re EEZ

Hello Tracy,
During the PacX mission we avoided foreign EEZs if possible. To the extent that it was necessary to transit through a foreign EEZ we do not believe the equipment and mission of the PacX Wave Gliders required prior approval from those jurisdictions. When we enter foreign territorial seas we do shut down all data collection devices other than those necessary for core communications and navigation.
Thanks,
Luke
